# Sustainable Thin-Film Composite Mixed-Matrix Membranes Based on Cellulose Acetate, Bimetallic ZIF-8-67, and Ionic Liquid for Enhanced Propene/Propane Separation

**DOI:** 10.3390/polym18030396

**Published:** 2026-02-02

**Authors:** Pegah Hajivand, Mariagiulia Longo, Marcello Monteleone, Alessio Fuoco, Elisa Esposito, Teresa Fina Mastropietro, Javier Navarro-Alapont, Donatella Armentano, Johannes Carolus Jansen

**Affiliations:** 1 CNR Institute on Membrane Technology “Enrico Drioli”, CNR-ITM, Via P. Bucci 17/C, 87036 Rende, CS, Italy; pegahhajivand@cnr.it (P.H.);; 2 Chemistry and Chemical Technology Department, Università della Calabria, Via P. Bucci 14/C, 87036 Rende, CS, Italy; 3 Departamento de Química Inorgánica, Instituto de Ciencia Molecular (ICMOL), Universidad de Valencia, 46980 Valencia, Spain

**Keywords:** cellulose acetate, ionic liquid, thin-film composite, mixed-matrix membranes, metal–organic frameworks, zeolitic imidazolate framework, gas separation, propane, propene

## Abstract

Efficiently separating propene and propane is paramount for the chemical industry but notoriously difficult due to their minimal size and volatility differences. Here, an efficient strategy to overcome this separation challenge was demonstrated through the design of bimetallic zeolitic imidazolate framework (ZIF)-based mixed-matrix membranes (MMMs). Thin-film composite (TFC) membranes were fabricated by integrating monometallic ZIF-8, ZIF-67, and a synergistic bimetallic ZIF-8-67 into a uniquely formulated ionic liquid–cellulose acetate (IL–CA) polymer matrix. Structural and morphological analyses confirmed the high crystallinity of the ZIF fillers and their seamless integration within the polymer. The resultant ZIF-8-67/IL-CA membrane exhibited notable separation performance, surpassing its monometallic counterparts by a threefold increase in both C_3_H_6_ permeance and C_3_H_6_/C_3_H_8_ ideal selectivity relative to the base membrane. Under industrially relevant mixed-gas testing, the membrane achieved a competitive separation factor of eight for propene over propane. These findings reveal that the strategic integration of bimetallic nodes in ZIFs can unlock synergistic properties unattainable with single-metal frameworks. This work presents a robust and scalable platform for developing next-generation membranes that defy conventional performance trade-offs, paving the way for efficient membrane-based olefin/paraffin separations.

## 1. Introduction

In 2024, the global propylene market size accounted for USD 112.02 billion, and it is anticipated to reach around USD 162.03 billion by 2034, with a 3.76% CAGR growth during the decade [1]. Typically, in industrial production, three main conventional methods are used to purify light hydrocarbons, namely distillation, extraction, and absorption, with steam cracking, followed by cryogenic distillation, being the most common technique [2]. Nevertheless, due to the very close molecular size and similar physical properties of C_3_H_6_ and C_3_H_8_ molecules (Table 1), it is very challenging, energy-intensive, and economically demanding to exploit cryogenic distillation for this separation [2,3]. To tackle it, alternative separation technologies, including membrane, absorption, and adsorption, have been suggested since the 1990s [4]. Among them, membrane-based technologies have recently provided promising results for various industrial gas separation processes, demonstrating high potential to considerably reduce the cost and energy demands compared to common methods [5,6].

Mixed-matrix membranes (MMMs) are a promising type of membranes in which suitable fillers are incorporated into a polymer matrix to fabricate a composite material. Among the different nanoporous filler materials, metal–organic frameworks (MOFs) have gained attention as a leading class of porous materials for various gas adsorption and separation applications and for the development of functional membranes, owing to their exceptional intrinsic features [8]. MOF reticular chemistry allows the prospective fabrication of porous materials with an elevated structural predictability [9]. The rational selection of suitable metal ions and organic linkers enables the precise control of pore size and shape at the Å scale, providing different functionalities and active sites as nodes and edges, and enhancing the flexibility of the MOF scaffold. Given the complexity of propene/propane separation, the results obtained with MOF-based materials, including inorganic MOF membranes [10,11,12,13], as well as MOF-based mixed-matrix membranes [14,15,16,17,18,19,20,21], are very promising.

**Figure 1:**
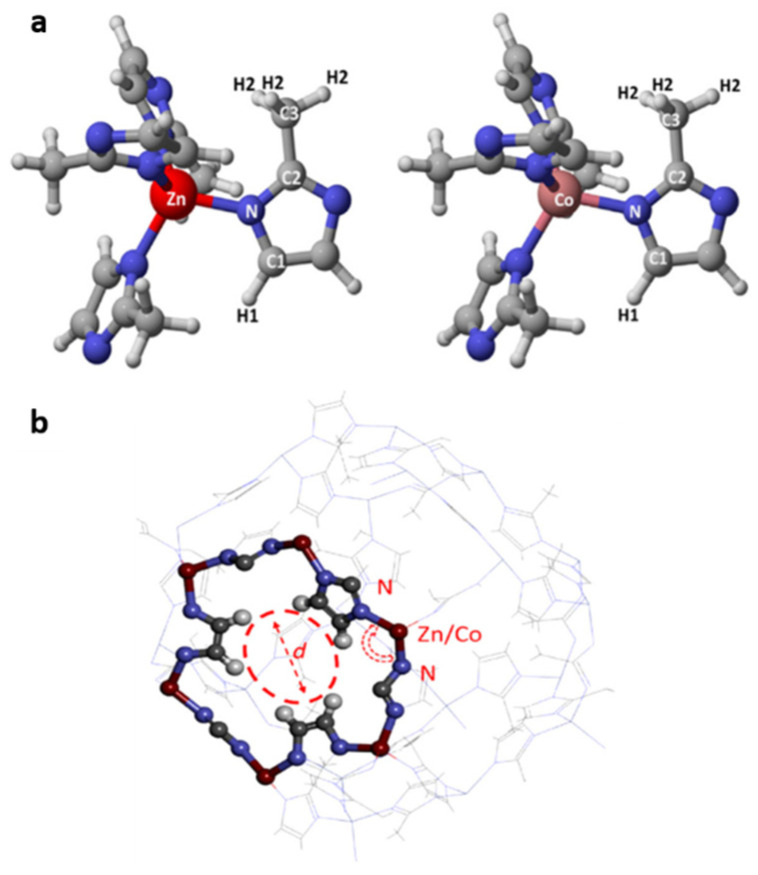
(**a**) Basic tetrahedral unit of ZIF-8 and ZIF-67 frameworks and (**b**) aperture leading to the cage of ZIF-8-67. The N–Zn/Co–N angle flexibility governs the aperture size response in the model. (Reprinted with permission from [26]. Copyright © 2016, American Chemical Society.).

Zeolitic imidazolate frameworks are among the first MOFs investigated for propene/propane separation, particularly the prototypical ZIF-8 [22,23,24], for which an ideal propene/propane selectivity of around 130 [22] was predicted. This highly selective performance originates from the appropriate effective aperture size of ZIF-8, from 4.0 to 4.2 Å, lying between the kinetic diameters of propene and propane [23]. Additional investigations revealed that the replacement of Zn ions in ZIF-8 with Co ions, resulting in ZIF-67 [25], clearly improves propene/propane separation, which was estimated to achieve the ideal selectivity of ~200 [26]. Computational techniques assessed that the presence of Co(II) in ZIF-67 results in a tighter structure with reduced flexibility of the pore opening, due to the robust bonding of Co with the N atoms of the ligands, leading to a narrower aperture size (Figure 1) [25]. ZIF-8 and ZIF-67 have also shown promising propene/propane separation when combined with different polymers to fabricate mixed-matrix membranes (MMMs) [14,16,17,27,28]. For instance, a MMM with 20 wt.% ZIF-67 filler in 6FDA-DAM as the polymer matrix showed a two-fold increase in C_3_H_6_/C_3_H_8_ selectivity compared to the ZIF-8 counterpart [29].

Hybrid ZIFs with mixed metal centres and/or mixed linkers were prepared and investigated towards C_3_H_6_/C_3_H_8_ separation [19], with CoZn-ZIF-8 inorganic membrane (Co/Zn ratio of 1) showing a separation factor of 120.2, with a remarkable 90% improvement compared to monometallic ZIF-8 (~63) and only a minor loss in C_3_H_6_ permeance (ca. 5%). Recently, ZIF-8-67 was used to fabricate 6FDA-DAM/ZIF-8-67 (70/30 wt.%) MMMs [30], showing substantially improved C_3_H_6_ permeability by up to 240% increase, and moderate C_3_H_6_/C_3_H_8_ selectivity enhancement (70%) compared to the pristine 6FDA-DAM membrane. This superior performance of the bimetallic CoZn-ZIF compared to its parent monometallic ZIFs has also been reported for other gas pairs, such as CO_2_/CH_4_ [31], CO_2_/H_2_ [32], and CO_2_/N_2_ [33].

Despite these advances, conventional solvothermal synthesis of ZIFs relies on organic solvents, raising concerns related to flammability, cost, and toxicity. To address these issues, ZIF-8 has recently been synthesised from aqueous solutions, either hydrothermally or at room temperature [34,35,36,37], using small amounts of additives or non-ionic surfactants as structure-directing agents [38,39,40]. Building on the demonstrated advantages of bimetallic ZIFs for propene/propane separation and the growing emphasis on green synthesis strategies, the integration of high-performance fillers with sustainable polymer matrices represents a promising route toward environmentally responsible MMMs. In this context, biopolymers offer an attractive platform to further reduce the environmental footprint while maintaining competitive separation performance.

Cellulose acetate (CA) is one of the first and the most reported bio-based polymers for gas separation, stemming from its non-toxicity, biodegradability, ease of availability [41,42,43], functional adjustability [44,45], high chemical and mechanical stabilities [41], and good gas-transport properties [46,47]. Nevertheless, due to its hydrophilic nature, crystallinity, and trade-off behaviour [48], as well as low hydrocarbon permeability and low diffusivity of larger molecules like C_3_ hydrocarbons [49], only a few reports explored this material for C_3_ separation [49,50,51,52,53]. In our recent work, we tailored the CA properties by incorporating appropriate ionic liquids (ILs) [54]. The obtained results showed that 30% *w*/*w* [BMIM]^+^[Tf_2_N]^−^ could effectively plasticise the polymer, enhancing the overall C_3_ permeability while maintaining a sufficiently high stiffness and mechanical strength to preserve the propene/propane selectivity. Having that success in mind, to further improve the separation of propene/propane, in the current study, either monometallic ZIFs (ZIF-8 and ZIF-67) and bimetallic ZIF (ZIF-8-67) were incorporated into IL-CA MMMs. Moreover, the membranes were prepared in the form of thin-film composites (TFCs) to address the long measurement time and low permeation coefficients of C_3_ hydrocarbons, particularly propane. The novelty of this work lies in the strategic combination of a multivariate (MTV) ZIF-8-67 with an IL-modified CA matrix, which exploits the combined effects of MTV-ZIF intrinsic selectivity and IL-assisted interfacial defect mitigation for the separation of C_3_H_6_ and C_3_H_8_. To the best of our knowledge, comprehensive single-gas and mixed-gases transport properties of this combination of materials, especially as TFCs with true scale-up potential, have not been studied before. Our findings demonstrate the superior separation performance of ZIF-8-67-based MMM, which achieves the highest C_3_H_6_/C_3_H_8_ selectivity compared to its monometallic parents.

## 2. Materials and Methods

### 2.1. Materials

Cellulose acetate (CA), with a degree of substitution (DS) of 2.34 and Mw = 92.0 kg mol^−1^ [54] was kindly provided by SNIA (Pisticci, Italy). Cobalt nitrate hexahydrate (Co(NO_3_)_2_·6H_2_O, 98%, Sigma-Aldrich, St. Louis, MO, USA), Zinc nitrate hexahydrate (Zn(NO_3_)_2_·6H_2_O, 98%, Sigma-Aldrich), 2-methylimidazole (C_4_H_6_N_2_, 99%, Sigma-Aldrich), and Triethanolamine (TEA) (98%, Merck, Darmstadt, Germany) were employed for the synthesis of ZIF-8, ZIF-67 and ZIF-8/67 without further purification.

The solvents acetone (98%), n-hexane (99.5%), methanol (99%), ethanol (99%), and isopropanol (98%) were purchased from Merck (Darmstadt, Germany). The ionic liquid 1-butyl-3-methylimidazolium bis (trifluoromethanesulfonyl) imide ([BMIM]^+^[Tf_2_N]^−^, C_10_H_15_F_6_N_3_O_4_S_2_), 98%, was supplied by Merck (Darmstadt, Germany) and Aldrich (St. Louis, MO, USA). Two-component silicone resin Elastosil^®^ M4601 (for post-treatment of the membrane defects) was provided by Wacker Chemie AG (Munich, Germany). Membrane supports of Polyacrylonitrile (PAN) and Polytetrafluoroethylene (PTFE, Teflon) were supplied by Deltamem (Allschwil, Switzerland) and Pall Italia Srl. (Buccinasco, Italy), respectively. To carry out the permeation tests, a series of gases, including N_2_, CH_4_, CO_2_ (purity of 99.99+%) and C_3_H_6_, C_3_H_8_ (purity of +99.5%), were obtained from Sapio (Monza, Italy).

### 2.2. MOF Synthesis

**Scheme 1:**
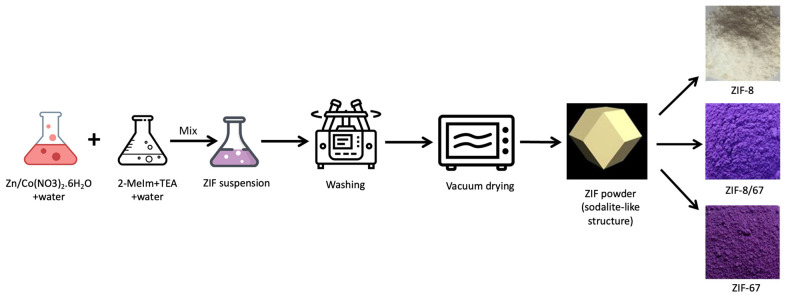
Schematic of the green synthesis of the ZIF nanoparticles.

ZIF-8, ZIF-67, and ZIF-8-67 were synthesised according to a green method [38], using a metal/ligand/TEA molar ratio of 1:8:8, with a modified washing procedure. To prepare ZIF-67 and ZIF-8, in the first step, Co(NO_3_)_2_·6H_2_O (0.7170 g) and Zn(NO_3_)_2_·6H_2_O (0.7361 g), respectively, were dissolved in 50 mL of deionised (DI) water under stirring for 20 min. In the case of ZIF-8-67, a mixed Co/Zn ion solution was made by dissolving Co(NO_3_)_2_·6H_2_O (0.7170 g) and Zn(NO_3_)_2_·6H_2_O (0.7330 g) in the same quantity of deionized (DI) water, following the same procedure as for ZIF-8 and ZIF-67. In the second synthetic step, which was identical for all ZIFs, a solution was prepared by mixing 1.622 g of 2-methylimidazole (2-MeIm), 2.00 g of TEA, and 50 mL of DI water, and stirring for 20 min until a uniform solution was obtained. Following this, for each ZIF (ZIF-8, ZIF-67, and ZIF-8-67), the corresponding solution of salt (nitrate of Zn^2+^, Co^2+^, or Zn^2+^/Co^2+^) was added to the solution of 2-MeIm and TEA, which resulted in changing the colourless solution to opaque white, opaque purple, and opaque bluish-purple suspensions, respectively. After a further 20 min of stirring, the mixtures were centrifuged (20 min at 6000 rpm), and the supernatant was decanted. Then, the remaining solids were washed using a group of solvents (water, methanol, ethanol, isopropanol, and acetone) to gradually decrease the polarity of the solvent and achieve a more uniform and consistent mixture with the polymeric solution (CA in acetone). Finally, the ZIF nanoparticles were resuspended in acetone and kept in a sealed jar for further usage (suspension I). For the physical characterisation (PXRD, porosimetry, FT-IR, ICP-MS, and SEM microanalysis), the ZIF suspension I was filtered on a filter paper before vacuum drying at 60 °C for 12 h. The dried powders were then weighed and stored in sealed jars for further use. Scheme 1 schematically illustrates the synthesis.

### 2.3. Membrane Preparation

Two series of MMMs were prepared. First, the CA polymer powder was dried at 100 °C overnight and then dissolved in acetone to obtain a 10 wt.%. solution of CA (solution I). The solution was magnetically stirred until homogeneous. For the first set of membranes, the ZIF-8 or ZIF-67 suspensions (suspension I) were mixed with the CA solution to obtain a 20 wt.%. of ZIF/CA mixtures (mixture I). Afterwards, the obtained suspensions were stirred for 24 h and sonicated for 8 h to obtain uniform mixtures.

**Scheme 2:**
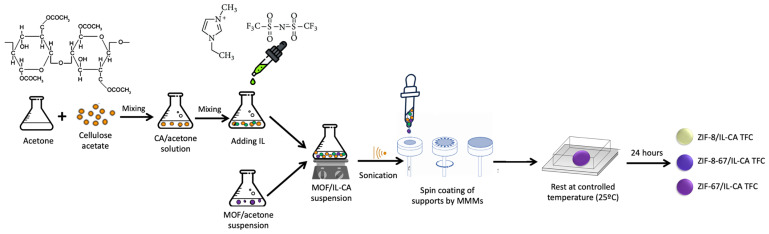
Schematic of the MMM preparation procedure by spin-coating.

For the second series of MMMs, [BMIM]^+^[Tf_2_N]^−^ ionic liquid was added to the polymer solutions. Motivated by the results obtained with the 30 wt.%. [BMIM]^+^[Tf_2_N]^−^-CA free-standing blended-membrane [54], the same IL with the same content was selected to form the second series of MMMs. A certain amount of the IL was added dropwise to the solution I to achieve the 30 wt.%. content, followed by stirring at ambient temperature overnight to obtain solution II. The suspension of either ZIF-8, ZIF-67, or ZIF-8-67 (suspension I) was mixed with solution II to achieve 20 wt.%. of ZIF/IL-CA. Following the same procedure as for the first MMM series, homogeneous suspensions of ZIF/CA-IL were obtained (mixture II). All the content ratios were calculated based on a dry weight basis, as Scheme 2 illustrates this preparation.

All polymeric solutions (solution I and solution II) and dispersions (mixture I and mixture II) were left for 2 h in the temperature-controlled chamber at 35 °C to degas before fabricating TFCs. A certain quantity of these samples was kept in a sealed jar to cast free-standing films for XRD and FT-IR analysis.

The first group of TFCs was prepared by the spin coating method. Here, to assess the effect of the support on the gas transport properties of the membranes, two different supports, PAN and PTFE, were selected. The first group of TFCs was fabricated by spin-coating at 1300 rpm solution I, mixture I, and mixture II (only ZIF-8 and ZIF-67 mixtures) on these two supports, with a homemade spin coater. A disc of the support (either PAN or PTFE), with a diameter of 4.7 cm, was placed in the spin coater. Then, somewhat less than 1 mL of pure acetone was dropped on the spinning support, immediately followed by dropping the same amount of the polymer/MOF solutions. Each support was coated twice (20 s of spinning and 20 s interval) with the corresponding mixture before placing it in the temperature-controlled chamber at 25 °C for drying overnight.

The second group of TFCs was produced, employing the same procedure with solution I, solution II, and mixture II (all the ZIF mixtures) on only PAN support at 5000 rpm. The effect of the coating speed was evaluated by comparing the gas transport properties of the TFCs coated on PAN. Additionally, to avoid eventual defects, two series of each single TFC were prepared: one was post-treated by covering with a PDMS solution (20 wt.% in n-hexane) for successive gas permeation tests, while the samples for SEM analysis were kept uncovered. The schematic of this preparation is summarised in Scheme 2, and Table SI 1 lists all fabricated MMMs and their chemical structures.

### 2.4. Physicochemical Characterisations

The crystallinity of the samples was evaluated with a powder diffractometer (Brucker, D2 PHASER 2nd generation, Karlsruhe, Germany) with Cu-Kα radiation, λ = 1.54056 Å. The active surface area and the average pore diameter of the synthesised ZIFs were characterised by BELSORP MINI X, Osaka, Japan. The samples were activated at 473.15 K (200 °C) for 16 h under reduced pressure, before carrying out the N_2_ adsorption−desorption isotherms and Brunauer–Emmett–Teller model (BET) analyses. Moreover, for SEM/XPS and ICP-MS analyses of the ZIFs, a SEM-HITACHI 4800 instrument (Tokyo, Japan) and an Agilent 7900 ICP-MS (Santa Clara, CA, USA) were employed, respectively. For SEM characterisation, the powder samples were mounted on electrically conductive carbon tape, without sputter-coating with gold, to ensure proper imaging and results. The ICP-MS analysis involved a preliminary microwave digestion of the powdered samples to prepare them for measurement, which was performed by the Microanalytical Service of the Universitat de València. FT-IR measurements were carried out using a Thermo Fisher iS50 FT-IR spectrometer with an ATR diamond (Waltham, MA, USA). All spectra were obtained from 64 scans with a resolution of 4 cm^−1^, in the range of 400–4000 cm^−1^. All characterisations were performed twice. Finally, the morphology of the TFCs was characterised by Scanning Electron Microscopy analysis, SEM, using Phenom Pro X desktop SEM, Phenom-World (Eindhoven, The Netherlands). TFCs were delicately cut after immersing the samples in liquid N_2_, and then the images were acquired with an accelerating voltage of 15 kV at different magnifications.

### 2.5. Gas Transport Properties

#### 2.5.1. Fixed-Volume Single-Gas Permeation Analyser

Single-gas permeation tests were conducted using a fixed-volume pressure increase device, designed by HZG and constructed by Elektro & Elektronik Service Reuter in Geesthacht, Germany, as previously described [55]. The tests were performed at a feed pressure of 1 bar and 25 °C, based on the time lag method. The membranes were equipped with an effective area of 13.84 cm^2^ and were fixed in a permeation module with two separate compartments (feed and permeate). Before exposing the membrane to a certain gas, the membrane was subjected to an evacuation of both sides of the membrane for a sufficient time to remove all previously absorbed species. Afterwards, from the feed side, the membrane is subjected to a series of gases, including N_2_, CH_4_, CO_2_, C_3_H_6_, C_3_H_8_, and the pressure is recorded on the permeate side with constant volume. The details of the time lag method are discussed in Section SI3.1.

#### 2.5.2. Mixed-Gas Permeation

Mixed-gas permeation analysis was conducted by a constant pressure/variable volume system equipped with a quadrupole mass filter (HPR-20 QIC Benchtop residual gas analysis system, Hiden Analytical, Warrington, UK). The mass spectrometric gas analyser enables continuous monitoring of individual species present in the gas mixture. The measurements were carried out using a mixture of C_3_H_6_/C_3_H_8_ containing 50 vol% of each gas, with a feed flow rate of 50 cm^3^ STP min^−1^ and using argon as the sweeping gas and internal standard for the gas analyser. Further details of the equipment were reported previously [55,56]. In Section SI3.2, the details of the applied method for determining permeability and diffusion coefficient (D) of the mixed-gas permeation are discussed.

## 3. Results and Discussions

### 3.1. Structural Analysis of ZIFs and MMMs

#### 3.1.1. ZIF Porosimetry Analysis

MOF surface area, pore size, and pore volume [57] were determined by the Brunauer–Emmett–Teller (BET) theory [58]. The surface area and porosity characteristics of the synthesised ZIFs are available in Figure SI 1 and Table SI 2. The N_2_ sorption–desorption isotherms for ZIF-8, ZIF-67, and ZIF-8-67 followed the type I isotherm (Figure SI 1a,c,e), indicating a microporous structure. In all ZIFs, the coexistence of micro- and mesoporosity in the bulk of the nanoparticles is confirmed by the first N_2_ uptake at very low pressures and the second rise in uptake, respectively [34]. Comparing the BET plots of the ZIFs (Figure SI 1b,d,f), all the ZIFs show a linear region at *P*/*P*_0_ = 0.05–0.3, confirming a uniform adsorption behaviour and a well-activated porous structure. The values of surface area, total pore volume, and average pore diameter for the synthesised ZIFs are also compared with those reported in the literature (Table SI 2). It indicates a noticeably higher BET surface area of this work’s ZIF-8 than what was achieved by the same method (976.5 and 620 m^2^·g^−1^, respectively) [38], comparable with what was synthesised via solvothermal methods, 962 m^2^·g^−1^ [34,59]. Similarly, our ZIF-67 shows a higher surface area than that reported in the literature for ZIF-67 prepared with the same synthesis protocol (1098 and 636 m^2^·g^−1^, respectively) [38], and slightly higher than the values reported for ZIF-67 prepared by employing higher amounts of TEA and water as solvent (1068 m^2^·g^−1^) [60]. This enhancement in active surface area could be attributed to the difference in the applied washing treatment, performed with a series of solvents, which can effectively eliminate pore-blocking residues, as well as to the presence of both micro- and mesoporosity or the reduced particle size of the crystal particles.

Comparing ZIF-8 and ZIF-67, the active surface areas are analogous, with ZIF-67 slightly higher than that of ZIF-8 (1098 and 976.5 m^2^·g^−1^, respectively), with a smaller average pore size (4.05 and 4.54 nm, respectively). This very subtle difference might be attributed to the higher electronegativity of the Co^2+^ ion, which limits the flipping motion of 2-mIM organic ligands, thus resulting in a stiffer ZIF-67 structure, with a reduced effective aperture size [26,61]. In the case of ZIF-8-67, despite revealing a comparable active surface area with ZIF-8, the average pore diameter is significantly lower than that of its parent ZIFs, suggesting that the combination of Co^2+^ and Zn^2+^ ions could lead to a denser structure with smaller pore diameters in ZIF-8-67. Summarising the BET analysis, the microporous structures of the ZIFs and their high porosity are confirmed, making them good candidates, particularly for ZIF-8-67, to discriminate propene from propane through size-sieving.

#### 3.1.2. MOF Structural Microanalysis

Quantitative SEM microanalysis of the ZIFs, indicating both the kind and the percentage of the involved elements in the building units of each ZIF, is illustrated in Figure SI 2. As expected, ZIF-8 and ZIF-67 show the presence of single Zn^2+^ and Co^2+^ ions, respectively, along with other relevant components (C and N). Moreover, ZIF-8-67 features a similar contribution of Zn^2+^ and Co^2+^ ions (12.65 and 11.90 wt.%, respectively), according to the 1:1 (50–50 mol.%) ratio of these metals used in the synthesis. These results were confirmed by ICP-MS measurements (Table SI 3), which illustrate the dominant presence of Zn^2+^ and CO^2+^ ions in ZIF-8 and ZIF-67 samples, respectively, as well as the balanced contribution of these ions in the ZIF-8-67 framework. These observations demonstrate the successful synthetic approach and appropriate compositions of these green ZIFs.

### 3.2. Crystallinity Identification of MOFs and MMMs (X-Ray Diffractometer (XRD) Analysis)

**Figure 2:**
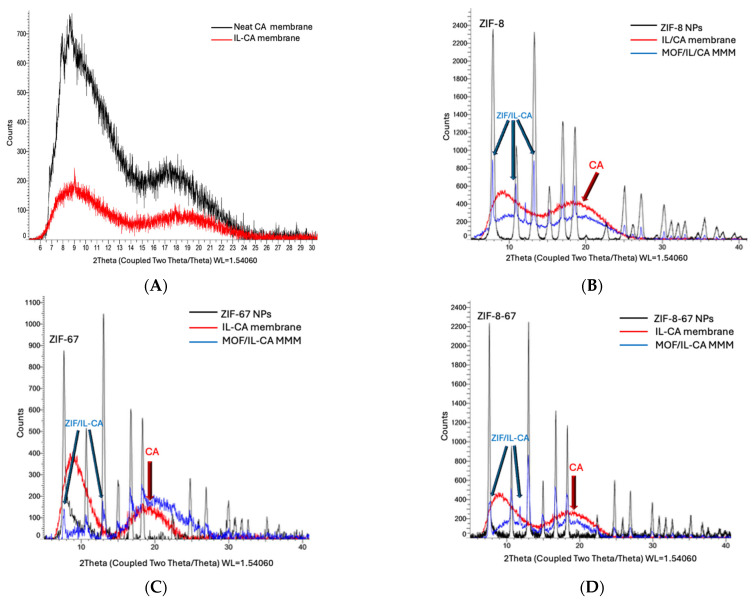
The XRD spectrum of (**A**) neat CA and IL/CA blended membrane, as well as its comparison with the MMMs containing (**B**) ZIF-8, (**C**) ZIF-67, and (**D**) ZIF-8-67.

Figure 2 shows the powder diffraction patterns of the polymeric membranes and ZIF-based MMMs. After adding 30% of [BMIM]^+^[Tf_2_N]^−^, the semi-crystalline structure of the neat CA membrane turned into an almost amorphous one (Figure 2A), as proved by the less intense and broadened peaks at 2θ = 8° and 18°, compared to the neat membrane. This phase transition was thoroughly discussed previously [54]. Figure 2B–D confirm that the crystallinity of ZIF-8 and ZIF-8-67 is fully preserved when included in the bulk of the amorphous IL-CA matrix, while ZIF-67 features a slightly reduced degree of crystallinity, as proven by the less sharp peak observed in the diffraction pattern of the corresponding MMM, which suggests a minor affinity between Co-containing ZIF and polymer during membrane preparation. The XRD spectra of the pure synthesised ZIF-8 and ZIF-67 are provided in Figure SI 3a,b, respectively. Both spectra were comparable with the reported simulated ones [62,63], confirming the accuracy of the performed diffraction and the purity of the synthesised materials. The XRD patterns of the as-synthesised ZIF-8 [64] and ZIF-67 [65] were compared with the standard JCPDS 00-062-1030 reference code, confirming their isostructural nature and phase purity. This reference, along with the simulated pattern from CCDC-671073, has been explicitly added to Figure SI 3. The main characteristic peaks of ZIF-8 are observed at 2θ = 6.53°, 11°, 13°, 15.5°, 17°,18.5°, 22.7°, and 25°, which agrees with the previous reports [66,67]. Both ZIF-67 and ZIF-8-67 powder diffraction patterns exhibit the major peaks at the same 2θ values as ZIF-8 (Figure SI 3c), with a minor right-shift at 2θ = 8°, 11°, and 14° for ZIF-8-67 with respect to its monometallic counterparts (Figure SI 3d).

### 3.3. Fourier Transform Infrared Spectroscopy (FT-IR) Analysis

The FT-IR spectrum of pure CA shows a distinctive peak at 1150 cm^−1^, attributed to the C–O–C asymmetrical stretching, at 1700 cm^−1^ and 1650 cm^−1^ for C=O symmetric and asymmetric stretching, respectively, and at 1300 cm^−1^ for δ–CH bending vibrations (Figure SI 4a, down), consistent with what was reported [68]. After adding the IL, [BMIM]^+^[Tf_2_N]^−^, new peaks in the range of 500–1500 cm^−1^ appeared, with the main vibration of the S=O group at 1173 (Figure SI 4a, top) [69]. In addition, peaks in the regions 830–835 cm^−1^ and 750–755 cm^−1^ are attributed to the C-H bending modes of the imidazolium ring [52,70].

Figure SI 4b shows the FTIR spectra of ZIF-8, ZIF-67, and ZIF-8-67, as pure powder, while Figure SI 4c shows the spectra of ZIF/IL-CA MMMs. The typical fingerprints of this ZIF family are at 1580 cm^−1^ (C=N stretch), and 1145 and 990 cm^−1^ (C–N and C–H stretch) with a relevant peak at 426 cm^−1^ related to Zn–N in ZIF-8, Co–N in ZIF-67, and Zn/Co–N in ZIF-8-67, in agreement with other studies [38,71]. The appearance of the new peaks in the ZIF/IL-CA spectra (Figure SI 4c) confirms that the fillers ([BMIM]^+^[Tf_2_N]^−^ and ZIF NPs) have been successfully included into the MMMs structure, in line with XRD results.

### 3.4. Membrane Fabrication and Characterisation

One of the key elements in fabricating a selective and defect-free TFC is the selection of a support possessing efficient porosity to both favour the permeation of the gases and prevent the penetration of the polymeric solution into the support during the coating process. The visual appearance of the TFCs, coated on PTFE at 1300 rpm and on PAN at 1300 and 5000 rpm, is shown in Figure SI 5. Comparing the TFCs, both PAN and PTFE supports appear homogeneously covered by the polymeric materials. However, the SEM images in Figure SI 6 of the neat CA, the ZIF/CA MMM, and the ZIF/IL-CA TFCs, coated at 1300 rpm, revealed that the PAN support provides better compatibility than PTFE with the neat CA. Microscopic observations indicated poor adhesion between the neat CA membrane and PTFE support and the existence of non-selective interfacial gaps, while the corresponding membrane on PAN support adheres uniformly. This could originate from the less hydrophobic nature of PAN and the possibility of forming hydrogen bonds and polar interactions between PAN and CA [72], which ensure good interfacial adhesion with minimum voids between the support and the membrane.

**Figure 3:**
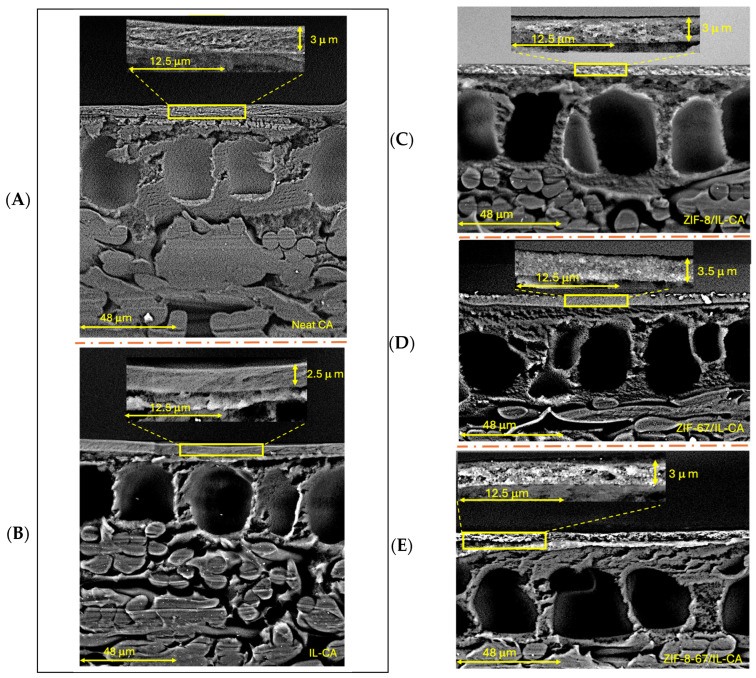
The SEM images of MMM TFCs coated at 5000 rpm on PAN. (**A**) neat CA, (**B**) IL-CA, (**C**) ZIF-8/IL-CA, (**D**) ZIF-67/IL-CA, and (**E**) ZIF-8-67/IL-CA. Magnification 1000× for the whole membrane and 3000× for the insert of the thin film when printed on A4-size paper.

The membranes coated at a higher speed (Figure 3) show a smoother and more uniform layer on the support with lower polymer/filler interfacial gaps, compared to those fabricated at 1300 rpm (Figure SI 6), particularly for ZIF/IL-CA MMMs. Moreover, the application of higher speed during the support coating resulted in TFCs with thinner skin, ranging from 2.5 to 3 µm, which is a key factor for enhancing the gas permeance, especially for larger molecules such as propene and propane through low-permeable polymers like the present CA. Coating at a sufficiently high speed also regulates the solvent evaporation rate via convection heat transfer and enhances the uniformity of the membrane-coated layer on the support surface [73,74]. Finally, the preliminary permeability results for the CO_2_/CH_4_ pair, in line with the SEM analysis, led to prioritising PAN support over PTFE for fabricating the TFCs; see Section SI3.2, Figures SI 8–SI 10 and Table SI 4.

### 3.5. Gas Transport Studies

#### 3.5.1. Single-Gas Permeation Properties of TFCs on PAN

##### CO_2_/CH_4_ and CO_2_/N_2_ Pairs

**Figure 4:**
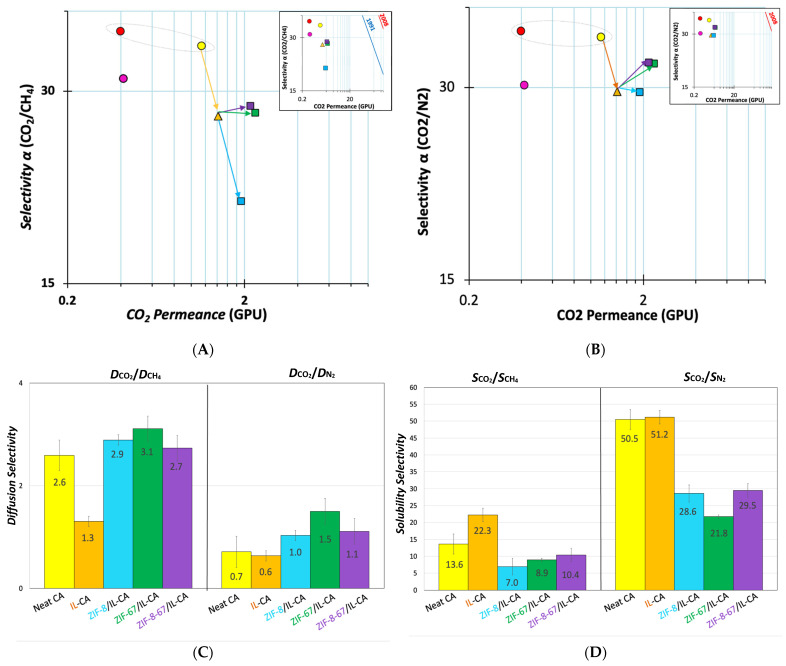
Robeson-like plots of TFC MMMs were prepared at 5000 rpm on PAN for (**A**) CO_2_/CH_4_ and (**B**) CO_2_/N_2_. Gas transport data of the neat polymer membrane are shown by circles (●), and the orange triangle (▲) represents IL-CA TFC. Squares (■) stand for ZIF/IL-CA TFCs (blue for ZIF-8, green for ZIF-67, and purple for ZIF-8-67). (**C**) The diffusivity selectivity of CO_2_/CH_4_ (**left panel**) and CO_2_/N_2_ (**right panel**), and (**D**) the solubility selectivity of CO_2_/CH_4_ (**left panel**) and CO_2_/N_2_ (**right panel**) are shown. Yellow is for neat CA, orange for IL-CA, blue for ZIF-8/IL-CA, green for ZIF-67/IL-CA, and purple for ZIF-8-67/IL-CA. The upper bound in the insert is shown for a hypothetical membrane, with a thickness of 1 µm. The blue line represents the 1991 upper bound and the red line represents the 2008 upper bound [81,82].

Robeson-like plots in Figure 4A,B compare the CO_2_/CH_4_ and CO_2_/N_2_ pair permselectivity of the TFCs fabricated at 1300 and 5000 rpm, and that of the free-standing film. The neat TFC at 5000 rpm presented three times higher CO_2_ permeance than both the free-standing membrane and the TFC at 1300 rpm (1.14 GPU), with higher CO_2_/CH_4_ selectivity (35.3) and CO_2_/N_2_ selectivity (36.8) than the free-standing film, 31.3 and 30.3, respectively. This demonstrates the benefits of fabricating TFCs at higher speeds, which yields thinner TFCs with higher gas permeance, and higher CO_2_/CH_4_ and CO_2_/N_2_ selectivity compared to the free-standing membrane.

Compared to the neat TFC at 5000 rpm, Figure 4A,B, [BMIM]^+^[Tf_2_N]^−^-CA TFC, show a slight increase in CO_2_ permeance but a decrease in CO_2_/CH_4_ and CO_2_/N_2_ ideal selectivity, reaching 27.4 and 29.6, respectively. Figure 4C,D display the solubility and diffusivity selectivity of CO_2_/CH_4_ and CO_2_/N_2_, respectively, calculated using the time lag method. It confirms that the IL reduced diffusivity selectivity for both gas pairs, with the opposite effect on solubility selectivity. Normally, the -OH groups in cellulose molecules possess a high affinity to absorb CO_2_ [75]. The groups could interact with the dipole moments of polar functional groups, such as fluoroalkyl groups in [BMIM]^+^[Tf_2_N]^−^, thereby inducing a dipole in the quadrupolar CO_2_ molecules. This interaction generally leads to higher absorbance and enhanced permeance of CO_2_ compared to CH_4_ and N_2_ [75], as reported in our previous study [54].

Adding the ZIFs increased the CO_2_ permeance from 1.41 for the neat polymer to 1.90, 2.30, and 2.15 for ZIF-8/MMM, ZIF-67/MMM, and ZIF-8-67/MMM, respectively, accompanied by a plateau in the ideal CO_2_/CH_4_ selectivity (~28), except for ZIF-8, compared to the CA-IL TFC (Figure 4A,B). In terms of CO_2_/N_2_ selectivity, while the ZIF-8/MMM maintained the value of the IL-CA TFC, ~28, ZIF-67/MMM and ZIF-8-67/MMM, by overcoming the trade-off obstacle, it slightly increased the ideal selectivity to ~33. Figure 4C,D show that the ZIFs generally increased the diffusivity selectivities compared to the IL-CA TFC due to molecular sieve-like properties. On the other hand, they decreased the solubility selectivities (Table SI 5). Overall, despite the enhancement in size-sieving separation of gases provided by the ZIFs, solubility selectivity dominated the CO_2_/CH_4_ and CO_2_/N_2_ separations. This is due to the higher affinity of ZIFs towards absorbing CO_2_ over CH_4_ and N_2_, enabling these MOFs to build both π- and σ-bonds with CO_2_, particularly in ZIF-67 and ZIF-8-67 due to the valence electrons of Co^2+^ (3d^7^) [76,77]. Similar findings, reported in other studies with ZIF-67/Pebax [78], ZIF-67/6FDA-Durene [79], ZIF-8/Matrimid^®^ [80], and bimetallic Zn/Co-ZIF/6FDA-ODA [31], support these results.

##### Permeation of the C_3_H_6_/C_3_H_8_ Gas Pair

**Figure 5:**
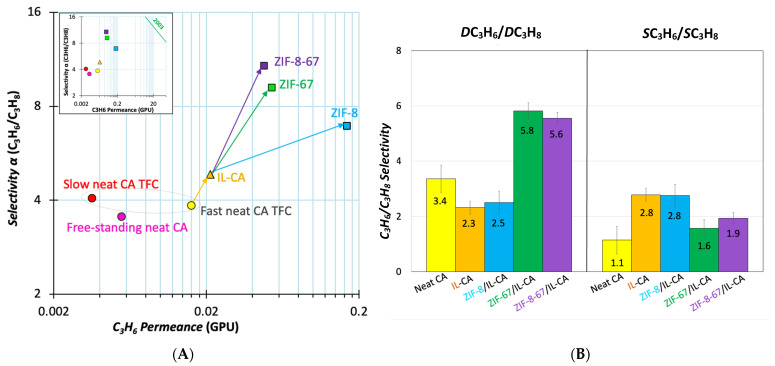
(**A**) Robeson-like plot for C_3_H_6_/C_3_H_8_ of CA-based MMMs with 30% content of the [BMIM]^+^ [Tf_2_N]^−^ and 20% ZIF-8, ZIF-67, and ZIF-8-67 on PAN; test area: 13.84 cm^2^. Gas transport data of neat membranes are shown by circles (●), and an orange triangle is used for IL-CA TFC at 5000 rpm (▲). Squares represent ZIF/IL-CA TFCs at 5000 rpm (■) (blue for ZIF-8, purple for ZIF-8-67, and green for ZIF-67). (**B**) The solubility selectivity (**left panel**) and diffusivity selectivity (**right panel**) of C_3_H_6_/C_3_H_8_ are depicted. The yellow column is for neat CA, orange for IL-CA, blue for ZIF-8/IL-CA, green for ZIF-67/IL-CA, and purple for ZIF-8-67/IL-CA. The upper bound in the inset is shown for a hypothetical membrane, with a thickness of 1 µm. The green line represents the 2003 upper bound [5].

Figure 5A displays an increased C_3_H_6_ permeance of the neat CA TFC prepared at 5000 rpm on PAN, which is three times and four times higher than that of the free-standing neat polymer film and the neat TFC prepared at 1300 rpm, respectively. Moreover, the neat TFC prepared at a higher speed showed a slight increase in C_3_H_6_/C_3_H_8_ selectivity compared to the free-standing film, from 3.5 to 3.9, which is comparable to the lower-speed made TFC. The IL-CA TFC at 5000 rpm also increased the C_3_H_6_ permeance of the neat CA TFC from 0.015 to 0.021 GPU, along with a rise in C_3_H_6_/C_3_H_8_ ideal selectivity from ~4 to ~5. While the plasticization effect of [BMIM]^+^[Tf_2_N]^−^ increases the C_3_ gases permeance, achieving higher ideal C_3_H_6_/C_3_H_8_ selectivity could be related to the higher affinity of this IL to interact with π-bonds of propene and absorb it over propane [54]. This hypothesis is confirmed by increasing the solubility selectivity but decreasing the diffusivity selectivity in IL-CA TFC compared to the neat CA TFC, depicted in Figure 5B and Table SI 5.

The incorporation of ZIFs further increased the propene permeance in the MMM TFCs, noticeably higher for the ZIF-8/MMM (0.17 GPU), along with a rise in the C_3_H_6_/C_3_H_8_ ideal selectivity to almost 2×, 2.5×, and 3× of the neat CA TFC (3.5) when using ZIF-8, ZIF-67, and ZIF-8-67, respectively (Figure 5A). The changes in the solubility and diffusivity selectivities (Figure 5B) demonstrate that the increase in the permeance of propene and the ideal C_3_H_6_/C_3_H_8_ selectivity in ZIF-67/ and ZIF-8-67/MMMs originates from the increase in the diffusivity difference in these gases rather than the solubility difference, while for ZIF-8/MMM both the solubility and diffusivity differences drove the separation.

##### Mechanistic Insight into ZIF-8, ZIF-67, and Synergistic Effect of Bimetallic ZIF-8-67 (MTV-ZIF)

The noticeable difference in the behaviour of ZIF/MMMs in C_3_ separation could be attributed to the synergistic properties of Zn^2+^ and Co^2+^. The higher electronegativity (EN) of the Co^2+^ ion (EN = 1.88), compared to that of Zn^2+^ (EN = 1.65) [76], results in a limited flipping motion of 2-mIM organic ligands; and therefore building stronger Co—N coordination bonds in ZIF-67 and a stiffer structure with a reduced effective aperture size [26,61]. A systematic study by An et al. [29] revealed that 20% ZIF-8 and ZIF-67 in 6FDA-DAM membranes altered the permeation activation energy, balancing diffusion activation energy (positive) and heat of sorption (negative) [83]. Judging from the higher energetic selectivity of ZIF-67 (375% more than ZIF-8) but its lower entropic selectivity (1.0 × 10^−10^, 71% less than ZIF-8), reported by Li et al. [24], based on the Transition State Theory (TST) model, An et al. attributed the ZIF-67 separation mechanism to the rigid structure of ZIF-67 which increases the repulsion energy that C_3_H_8_ must overcome to jump from one cavity to another. In addition, Cobalt-containing ZIFs, particularly ZIF-8-67, showed a narrower pore volume than ZIF-8 (Table SI 2), which enhances the size-sieving capability of these MOFs to discriminate larger molecules such as propane.

For ZIF-8-67/MMM, all observations (Figure 4 and Figure 5 and Table SI 5) suggest that this multivariant ZIF (MTV-ZIF) benefits from the properties of both ZIFs. Metals play a key role in guest adsorption and storage and allow tailoring of the ZIF pore size, a decisive tool for controlling host–guest interactions and separation ability [84,85]. In ZIF-8-67, the random co-location of Zn^2+^ and Co^2+^ creates chemically heterogeneous pores with a smaller average diameter than the parent ZIFs, giving higher ideal selectivity than pure ZIF-8 or ZIF-67. Moreover, Co^2+^ sites introduce chemical affinity towards C_3_H_6_ due to a slightly more polarising and/or electronegative metal centre compared to Zn^2+^, enhancing π-complexation or quadrupole-induced interactions with the C=C bond in propylene and boosting selectivity. On the other hand, Zn^2+^ sites provide more flexible apertures and weaker gas interactions. In the MTV framework, Zn^2+^ acts as a structural diluent for Co^2+^, moderating the overall interaction energy, resulting in Co^2+^ enhancing selective sorption, while the Zn^2+^ maintains low enthalpy for high kinetic accessibility (flux). Oh et al. embedded 20 wt.% of the same series of solvothermal ZIFs in 6FDA-DAM MMMs, tested at 2 atm and 35 °C for C_3_ separation. Compared to our findings, their ZIF-8-67 showed a higher aperture size compared to their corresponding ZIF-8 and ZIF-67, exhibiting the highest C_3_H_6_ permeability (69 Barrer) but the lowest C_3_H_6_/C_3_H_8_ selectivity (16.6) than ZIF-8/MMM and ZIF-67/MMM. This highlights the importance of obtaining NP fillers with smaller pores, which are highly effective for the yield of MMMs. Comparing their selectivity data (which increased from 11 for neat 6FDA-DAM to 18 for ZIF-8, 20 for ZIF-67, and 17 for ZIF-8-67 (representing 1.6×, 1.7×, and 1.5× increases, respectively) with our obtained results, it is evident that the final selectivity enhancement is higher for our series (rising from 3.8 for neat CA to 7 for ZIF-8, 9 for ZIF-67, and 11 for ZIF-8-67, corresponding to 2×, 2.4×, and 3× increases, respectively). The productivity of our introduced system is also proven when it showed higher C_3_ ideal selectivity compared to 20% ZIF-8@PPO-40 and ZIF-67@PPO-40 MMMs, with 1.3× and 1.6× increases, respectively [86], as well as compared to 20% ZIF-8/6FDA-Durene/DABA [21] and 20% ZIF-67/6FDA-DAM [87] with 1.55× and 1.45× increases in pristine membrane ideal selectivity, respectively.

#### 3.5.2. Propene/Propane Mixture Separation

The first challenge in the separation of propene/propane mixtures was the correct quantitative analysis of both gases. Since fragmentation of propane also produces propene, all further fragmentation causes overlap of the propene signals by propane signals, and, therefore, propene did not have any unique signals (Figure SI 7). Instead, propane has a unique signal at *m/z* = 29 amu, corresponding to the ethyl^+^ fragment, which is also the most intense signal in the mass spectrum of propane. The largest peak of propene is the signal at *m/z* = 41 amu, but this is too close to the extremely strong argon peak at 40 amu, and, therefore, the net signal of propene in the mixture is calculated from the peak at 42 amu as follows:

(1) $${I_{m/z\;42\;\left(propene\right)}=I}_{m/z\;42}-\left(\frac{I_{m/z\;29}\;\cdot \;\;2.53}{100}\;\right)$$

where the factor 2.53 is the correction for the relative intensity of the 29 amu peak related to the propene fragment of propane in the mixture. The propene concentration is then calculated by the procedure described in the experimental section and the Supporting Information. Overall, propane is usually the minor component in the mixture after permeation, and combined with its relatively small signal at *m/z* = 42 amu, its presence does not affect the accuracy of the propene determination, and thus the mixture composition can be determined reliably. To avoid signal overlap with the molecular ion tail of argon (*m/z* = 40), which serves as the sweep gas, argon was monitored respecting the ^36^Ar isotope at *m/z* = 36 amu, allowing for all signals to be analysed within a consistent range of partial pressure using the Secondary Electron Multiplier ion detector. In addition, measuring argon at its 0.3% isotope is advantageous because its signal intensity is closer to that of the other peaks, thereby improving the accuracy of the calculations.

**Figure 6:**
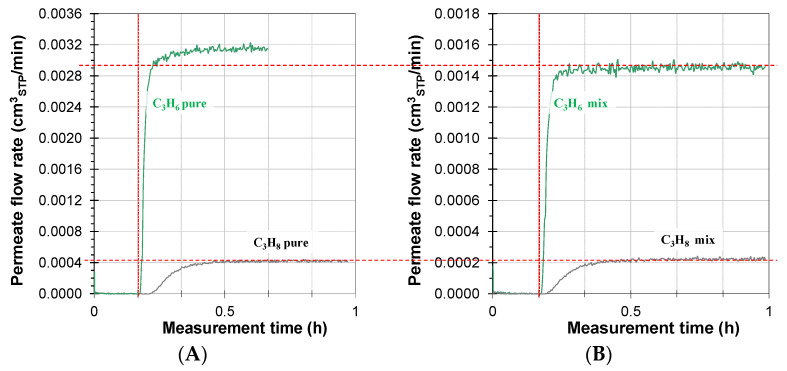
Propene and propane flow rate for pure gases (**A**) and as a 50/50 vol% mixture of propene and propane (**B**). The dashed vertical lines represent the start of the experiment, when the membrane is exposed to the gas. The dashed horizontal lines highlight the differences in signal.

The highest C_3_ selectivity of ZIF-8-67/IL-CA TFC and its reasonable propene permeance led to the selection of this TFC for further mixed-gas analysis. The permeation curves of pure propene and propane and their 50/50 vol% mixture are given in Figure 6A and Figure 6B, respectively. For a qualitative comparison, the scale of the mixture mode is expanded twice.

The pure propene flow rate is approximately eight times higher than that of propane, which confirms a high selectivity for the olefin species. A similar trend is observed for the gas mixture, but with a higher flow rate of propane and a lower propene flow rate, causing the lower mixed-gas selectivity than the ideal selectivity. This difference stems from the bulk effect caused by the high permeance of C_3_H_6_ in both the polymer and ZIF-8-67 cavities, facilitating the faster transport of larger gas molecules (C_3_H_8_) via carrier-mediated movement by smaller gas molecules (C_3_H_6_), thereby reducing separation factors under mixed-gas conditions [17]. A similar behaviour was observed in the permeability of propene and propane through ZIF-8/PIM-6FDA-OH MMMs, attributed to the differences in the sorption of C_3_H_6_ and C_3_H_8_ within both the polymer matrix and the molecular sieve, as well as the comparable condensability of C_3_H_6_ and C_3_H_8_, which induces strong negative sorption coupling effects between these gases [17].

**Figure 7:**
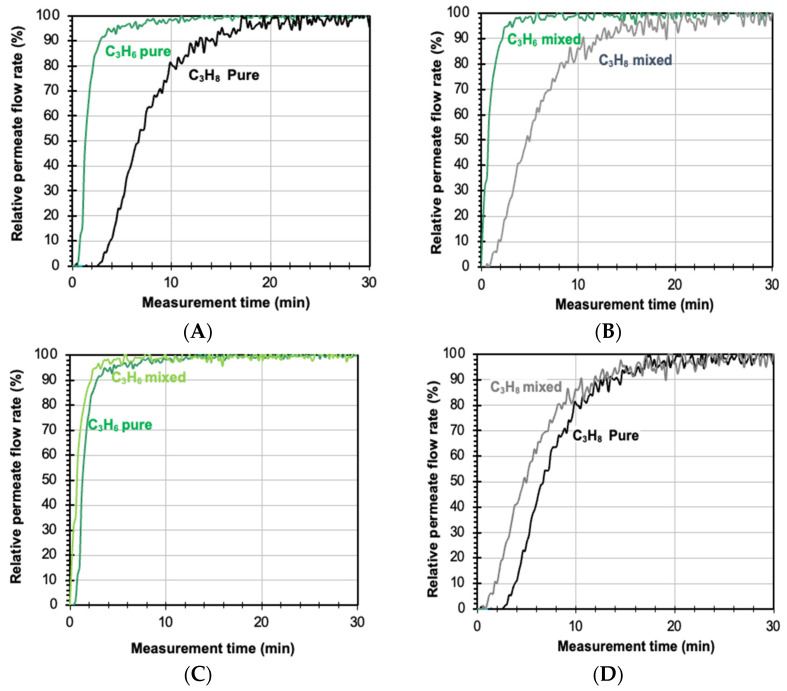
Normalised flow rates for pure gases (**A**) and mixtures (**B**), highlighting the large difference in diffusion coefficient of propene and propane. Comparison of the normalised pure and mixed-gas flow rate of propene (**C**) and propane (**D**), highlighting the virtually identical trends of the pure and mixed gases, with in both cases faster diffusion of the mixed-gas species. The x-axis is shown from the start of the experiment, when the membrane is exposed to the gas.

Interestingly, while the diffusion of light gases is too fast to observe a measurable time lag, a clear transient phenomenon is observable for propane and propene. Analogous to the traditional time lag curves in the fixed-volume setup for single gases, the S-shaped curve, due to the sensibly lower diffusion coefficient of the hydrocarbons than that of the light gases, can be used to evaluate the diffusion coefficient from the inflection point in the curve or from the time at half height [88,89]. The normalised flow rate, which highlights the differences between the individual gases, shows a much slower transient for propane than for propene, both in the single-gas mode (Figure 7A) and in the mixture mode (Figure 7B).

The diffusion coefficient of propene is much higher than that of propane due to its smaller effective diameter [90]. Direct comparison of the pure and mixed propene (Figure 7C) and propane (Figure 7D) shows that there is virtually no difference between the two propene curves, whereas propane is clearly much faster in the mixture than in the pure gas. This indicates a higher diffusion coefficient, which is thus at least in part responsible for the higher mixed-gas permeability of propane. The quantitative data are listed in Table SI 6. Since the precise membrane thickness is not accurately known, the absolute value of the diffusion coefficient cannot be determined, but the diffusion selectivity can still be determined from the ratio of the two-time lags, showing an increased diffusion selectivity from 5.6 to 8.6 (Table SI 6).

## 4. Conclusions and Outlook

This study comprehensively analyses MMMs, consisting of 20 wt.%. aqueous-based ZIFs (ZIF-8, ZIF-67, ZIF-8-67), in 30% [BMIM]^+^[Tf_2_N]^−^-CA blends, towards gas separation. The physical characteristics of ZIFs via BET analysis, XRD, SEM microanalysis, ICP-MS, and FT-IR revealed their highly crystalline and microporous structure, the successful approach of the aqueous-based synthesis of ZIFs, and the balanced presence of Zn^2+^ and Co^2+^ ions in ZIF-8-67, distinguishing it from its monometallic counterparts. The systematic investigation of TFC membranes fabricated at different coating speeds revealed key insights into their gas separation performance:

1. The transport properties of MMM TFCs on PAN and PTFE prioritised PAN support over PTFE.
2. Higher coating speeds led to the formation of thinner selective layers and enhanced gas diffusion, benefiting CO_2_ and C_3_H_6_ separations.
3. The presence of [BMIM]^+^[Tf_2_N]^−^ enhanced ZIF and CA compatibility, facilitated CO_2_ and C_3_H_6_ permeation, and increased C_3_H_6_/C_3_H_8_ ideal selectivity, while CO_2_/CH_4_ and CO_2_/N_2_ separation showed a trade-off behaviour.
4. The contribution of ZIFs further enhanced CO_2_ and C_3_H_6_ permeances, with ZIF-8-67 and ZIF-67 presenting the highest CO_2_/CH_4_ and CO_2_/N_2_ ideal selectivities. In C_3_H_6_/C_3_H_8_ separation, ZIF-8-67 exhibited the best ideal selectivity.
5. Mixed-gas separation experiments also confirmed a high selectivity for propene over propane via the ZIF-8-67/IL-CA TFC, demonstrating the practical relevance of this membrane.

Overall, this study highlights the significant role of bimetallic ZIF-8-67 in enhancing gas separation performance when incorporated into IL-CA membranes. The synergistic properties of Zn^2+^ and Co^2+^ in ZIF-8-67 and its small pore diameter, combined with the role of [BMIM]^+^[Tf_2_N]^−^ in reducing interfacial defects, contributed to its superior gas size-sieving effect and selectivity. These findings provide valuable insights for the development of advanced membranes for efficient propene/propane separation, paving the way for a scalable membrane that can be used at an industrial level.

The present work, committed to sustainable separation strategies, suggests using Bio MOFs and green bio-based ionic liquids for developing a new generation of sustainable MMM TFCs, which will be the subject of future work.

## Figures and Tables

**Table 1:** The physical properties of propene and propane [7].

	C_3_H_6_	C_3_H_8_
Molecular formula	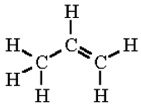	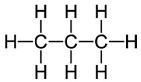
Molecular weight (g mol^−1^)	42.08	44.1
Normal boiling point (°C)	−47.69	−42.13
Kinetic diameter (nm)	0.45	0.43
Polarizability × [10^−25^ cm^3^]	62.6	62.9–63.7
Dipole moment × [10^18^ esu^−1^∙cm^−2^]	0.366	0.084
Critical temperature (°C)	91.75	96.74
Critical pressure (Bar)	45.55	42.51
Vapour pressure (Bar at (°C))	9.17 (21.1)	8.41 (21.1)

## Data Availability

The original contributions presented in this study are included in the article/Supplementary Material. Further inquiries can be directed to the corresponding authors.

## Supplementary Materials

The following supporting information can be downloaded at: https://www.mdpi.com/article/10.3390/polym18030396/s1. References [34,38,54,55,60,62,63,89,91,92,93] are cited in the supplementary materials. Figure SI 1: The N_2_ adsorption-desorption of (a) ZIF-8, (c) ZIF-67, (e) ZIF-8-67 and BET plots of (b) ZIF-8, (d) ZIF-67, (f) ZIF-8-67; Figure SI 2: Quantitive SEM microanalysis of the synthesised ZIFs (a) ZIF-8, (b) ZIF-67, (c) ZIF-8-67; Figure SI 3: (a) ZIF-8 and its simulated spectrum [5] (b) ZIF-67 and its simulated spectrum [6], (c) ZIF-8-67, (d) comparison of ZIFs; Figure SI 4: The FTIR spectra of (a) neat CA and IL-CA blend-membrane, (b) ZIF family, (c) ZIF/IL-CA MMMs; Figure SI 5: The physical appearance of all fabricated TFCs; Figure SI 6: SEM images of MMM TFCs made at 1300 rpm on PAN and PTFE. (a,e) neat CA, (b,f) ZIF-8/CA, (d,h) ZIF-67/CA, (c,g) ZIF-8/IL-CA, (e,i) ZIF-67/IL-CA at 1300 rpm; Figure SI 7: Electron ionization (EI) Mass spectrum for (a) propane and (b) propene; Figure SI 8: The CO_2_ permeance and CO_2_/CH_4_ selectivity through free-standing film and MMM TFCs on PAN and PTFE; Figure SI 9: CO_2_ permeation versus time for neat CA membranes (a) free-standing, (b) TFC on PAN at 1300 rpm, (c) TFC on PTFE at 1300 rpm; Figure SI 10: CO_2_ permeance versus time for TFCs, fabricated at 1300 rpm, on (a) PAN and (b) PTFE; Table SI 1. Chemical structures of the involved materials in MMMs; Table SI 2. The synthesized ZIF’s porosity data in comparison with other hydrothermal methods; Table SI 3. ICP-MS analysis results; Table SI 4. Pinhole effect on neat CA membranes (film and TFC) gas permeability measurement; Table SI 5. Gas transport properties of ZIF/IL-CA TFC MMMs on PAN at 5000 rpm; Table SI 6. Transport parameters of TFC membrane ZIF-8-67/IL-CA.
