# Supplementary material for: Sustainable Thin-Film Composite Mixed-Matrix Membranes Based on Cellulose Acetate, Bimetallic ZIF-8-67, and Ionic Liquid for Enhanced Propene/Propane Separation
Source: Polymers (Basel). 2026 Feb 2;18(3):396. doi: 10.3390/polym18030396 (PMC12899611; doi:10.3390/polym18030396)
Supplement: Supplementary file 1 [file polymers-18-00396-s001.zip › polymers-4069906-supplementary.pdf]

Supplementary Information

Sustainable Thin-Film Composite Mixed-Matrix Membranes Based on Cellulose Acetate, Bimetallic ZIF-8-67, and Ionic Liquid for Enhanced Propene/Propane Separation

Pegah Hajivand [1,2], Mariagiulia Longo [1], Marcello Monteleone [1], Alessio Fuoco [1], Elisa Esposito [1], Teresa Fina Mastropietro [2], Javier Navarro-Alapont [3], Donatella Armentano [2,*] and Johannes Carolus Jansen [1,*]

[a] Institute on Membrane Technology, CNR-ITM, Via P. Bucci 17/C, 87036, Rende (CS), Italy.

[b] Chemistry and Chemical Technology Department, Università della Calabria, Via P. Bucci 14/C, 87036, Rende (CS), Italy.

[c] Departamento de Química Inorgánica, Instituto de Ciencia Molecular (ICMOL), Universidad de Valencia, Valencia 46980, Spain

Index

SI1. Materials 2

SI2. Characterization 3

SI3. Transport properties 11

SI3.1. Fixed-volume single-gas Permeation Analyser 11

SI3.2 Mixed-gas Permeation 11

SI3.3. Single-gas permeation through MMMs on PAN and PTFE 13

SI1. Materials

Table S1. Chemical structures of the involved materials in MMMs

Membrane	support	Speed (rpm)	Structure
Neat CA	PAN	1300	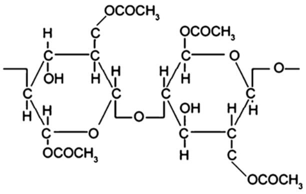
		5000	
	PTFE	1300	
ZIF-8/CA (20%wt.ZIF in CA)	PAN	1300	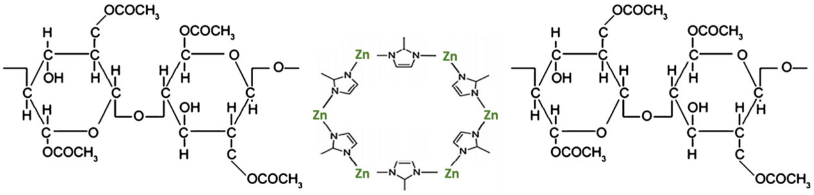
	PTFE	1300	
ZIF-67/CA (20%wt.ZIF in CA)	PAN	1300	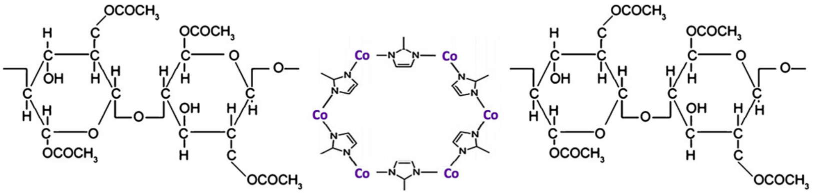
	PTFE	1300	
IL-CA blended-membrane (30%wt.IL in CA)	PAN	5000	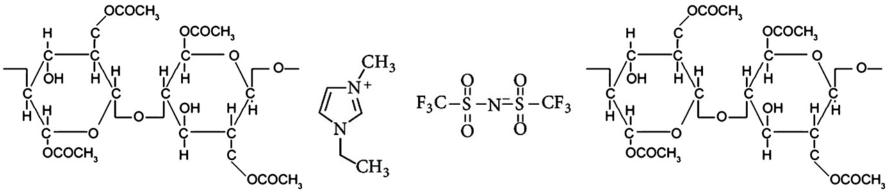
ZIF-8/IL-CA (20%wt.ZIF in IL/CA)	PAN	1300	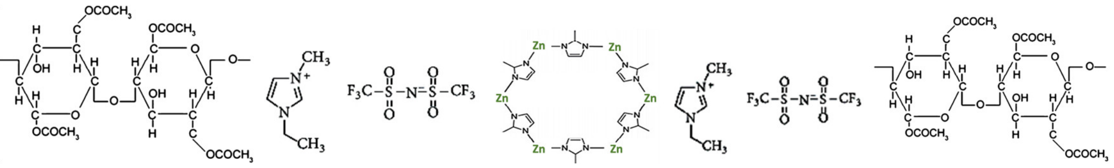
		5000	
	PTFE	1300	
ZIF-67/IL-CA (20%wt.ZIF in IL/CA)	PAN	1300	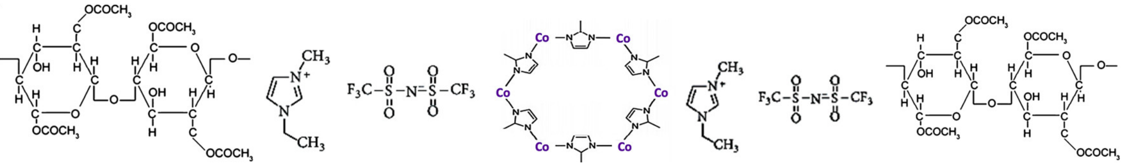
		5000	
	PTFE	1300	
ZIF-8-67/IL-CA (20%wt.ZIF in IL/CA)	PAN	5000	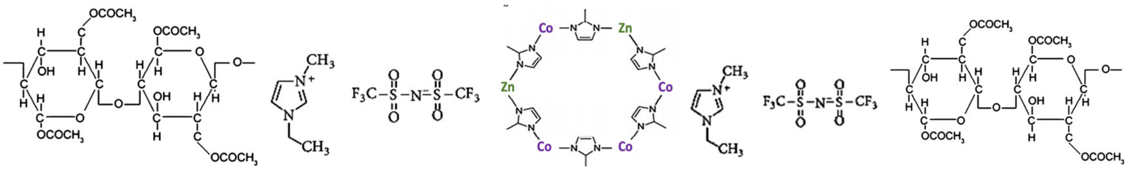

SI2. Characterization

Table S2. The synthesized ZIF's porosity data in comparison with other hydrothermal methods

MOF	Component Ratio [metal: ligand: TEA: H_2O]	Surface area (m^2g^{-1}) (BET)	Total pore volume (cm^3g^{-1})	Average pore diameter (nm)	Ref.
ZIF-8	1 : 8 : 8 : 2256	976.5	1.110	4.54	This work
	1 : 8 : 8 : 2256	620	-	-	[1]
	1 : 70 : 0 : 1238	1079	-	-	[2]
	1 : 6 : 2 : 500	491.5	-	-	[3]
	1 : 16 : 16 : 4512	1048	-	-	[4]
ZIF-67	1 : 8 : 8 : 2256	1098	1.114	4.05	This work
	1 : 8 : 8 : 2256	636	-	-	[1]
	1 : 16 : 16 : 4512	1068	-	-	[4]
ZIF-8-67	1 : 8 : 8 : 2256	903.1	0.611	2.70	This work
	(0.5 : 0.5) : 16 : 16 : 4512	1260	-	-	[4]

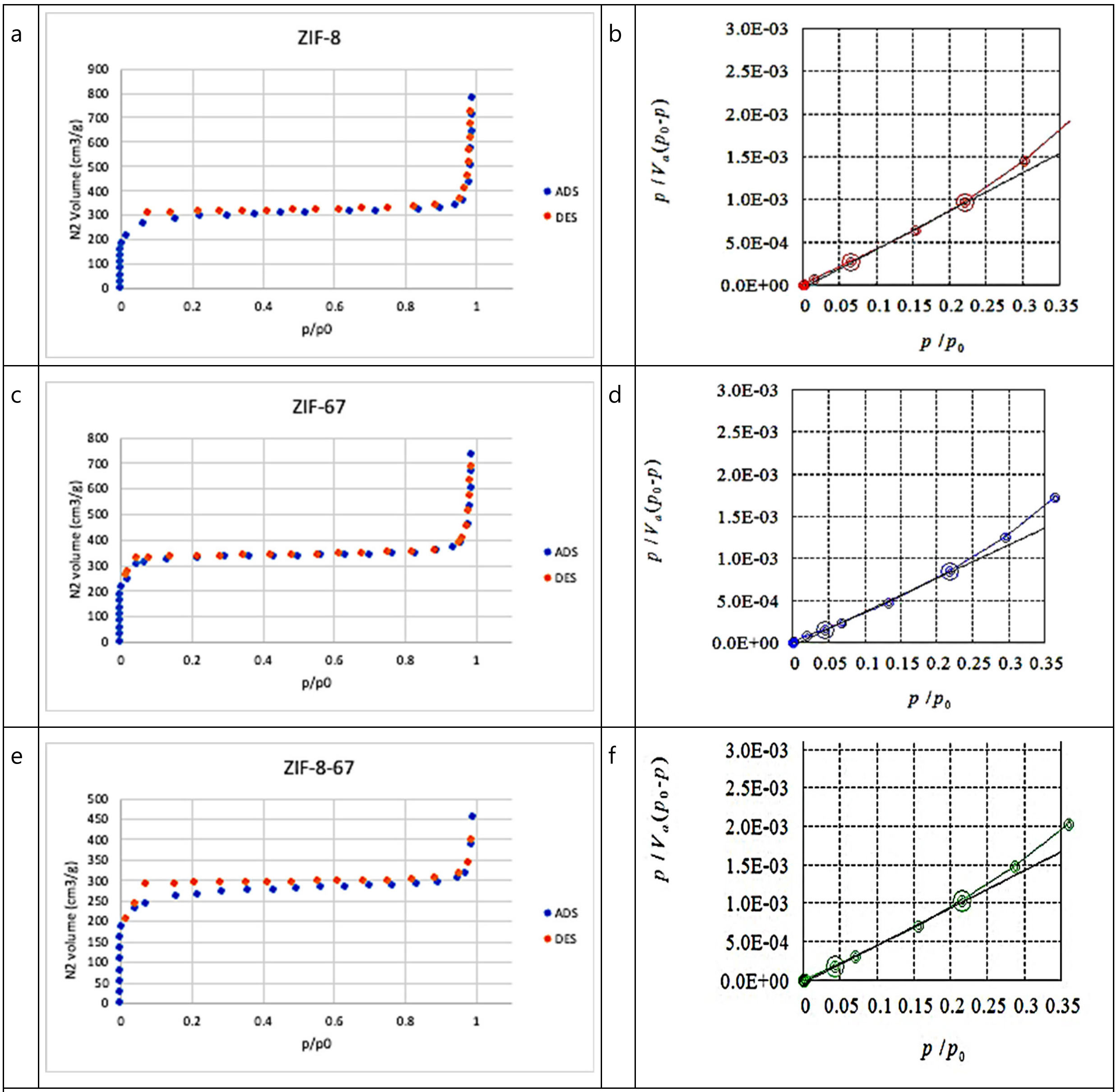

Figure S1. The N_2 adsorption-desorption of a) ZIF-8, c) ZIF-67, e) ZIF-8-67 and BET plots of b) ZIF-8, d) ZIF-67, f) ZIF-8-67.

a

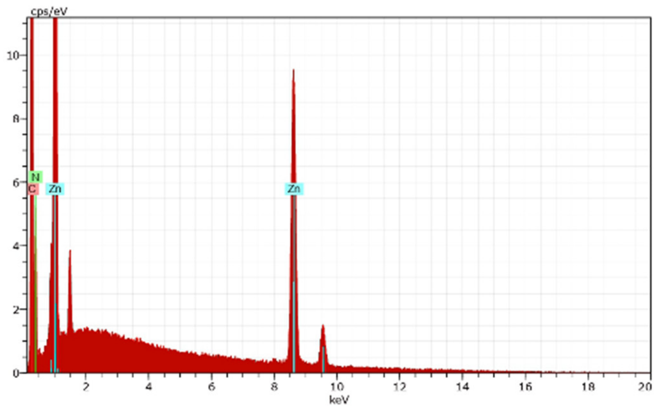

Component	Atom.C(at %)	Norm.C(wt %)
C	60.94	40.43
N	31.8	24.60
Zn	7.27	26.26

b

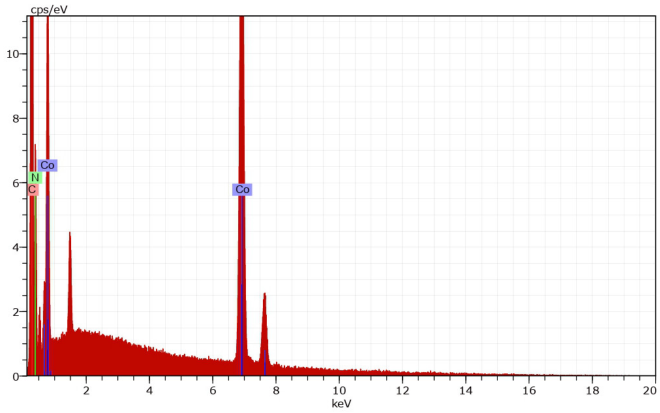

Component	Atom.C(at %)	Norm.C(wt %)
C	59.86	45.42
N	33.43	29.58
Co	6.72	25.01

c

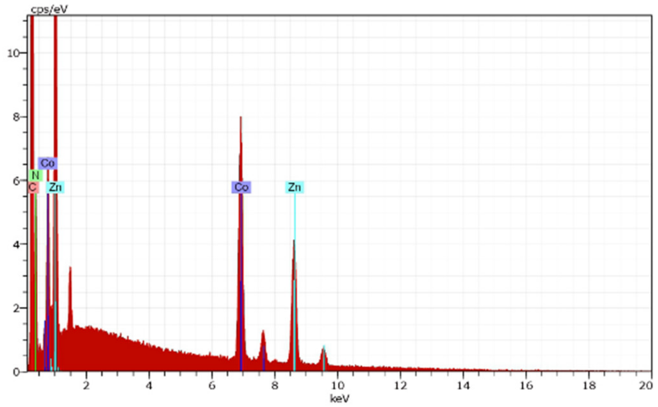

Component	Atom.C(at %)	Norm.C(wt %)
C	63.78	48.72
N	30.01	26.73
Zn	3.04	12.65
Co	3.17	11.90

Figure S2. quantitive SEM microanalysis of the synthesised ZIFs a) ZIF-8, b) ZIF-67, c) ZIF-8-67.

Table S3. ICP-MS analysis results

MOF	Co (mg/g)	Zn (mg/g)
ZIF-8	< 0.001	233 ± 3
ZIF-67	208 ± 2	0.194 ± 0.009
ZIF-8-67	106.9 ± 1.3	118.4 ± 1.2

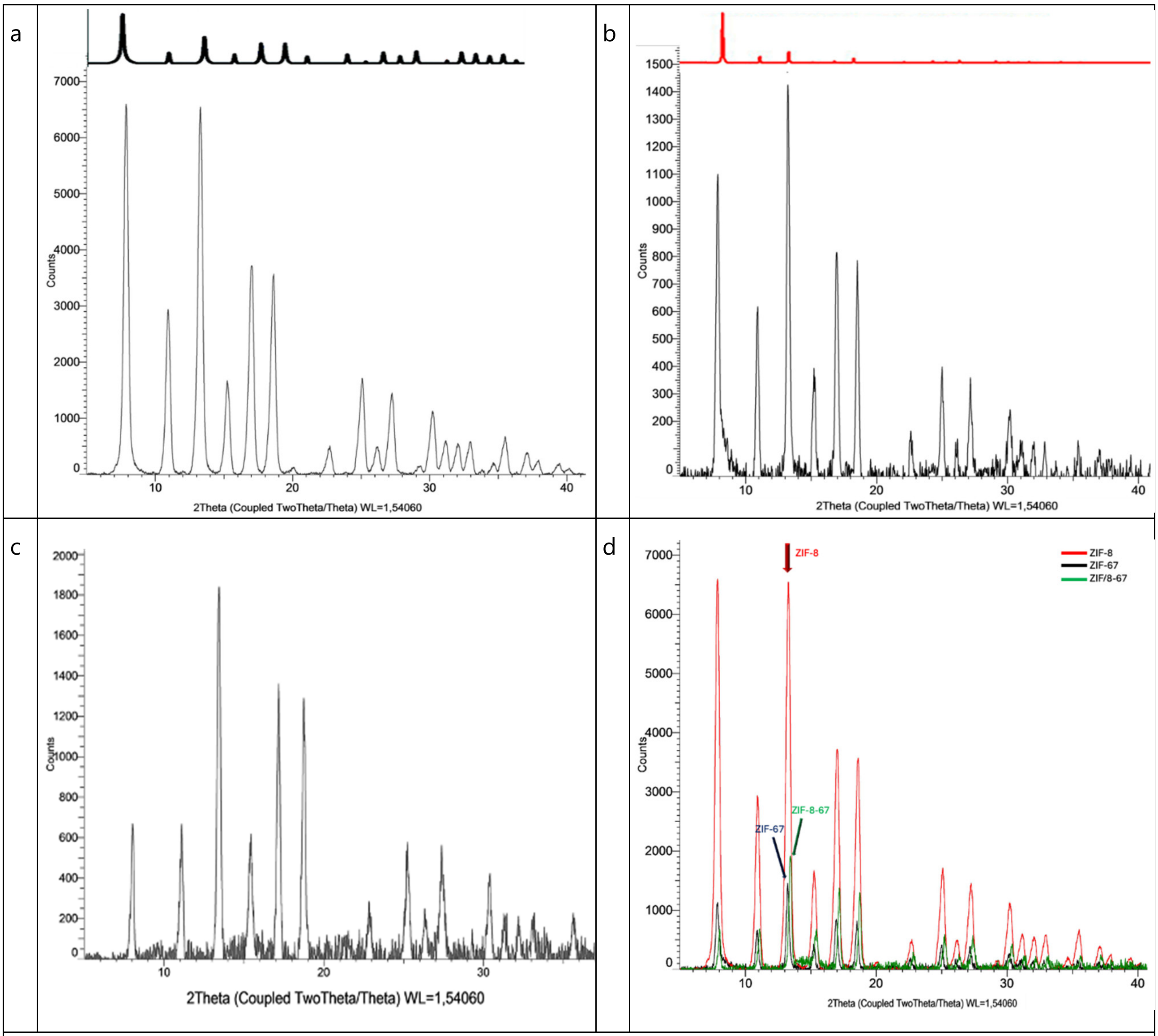

Figure S3. a) ZIF-8 and its simulated spectrum [5] b) ZIF-67 and its simulated spectrum [6], c) ZIF-8-67, d) comparison of ZIFs. JCPDS reference code of ZIF-8 and ZIF-67 is 00-062-1030 and the simulated pattern of these MOFs are available in Cambridge Structural Database (using CCDC-671073).

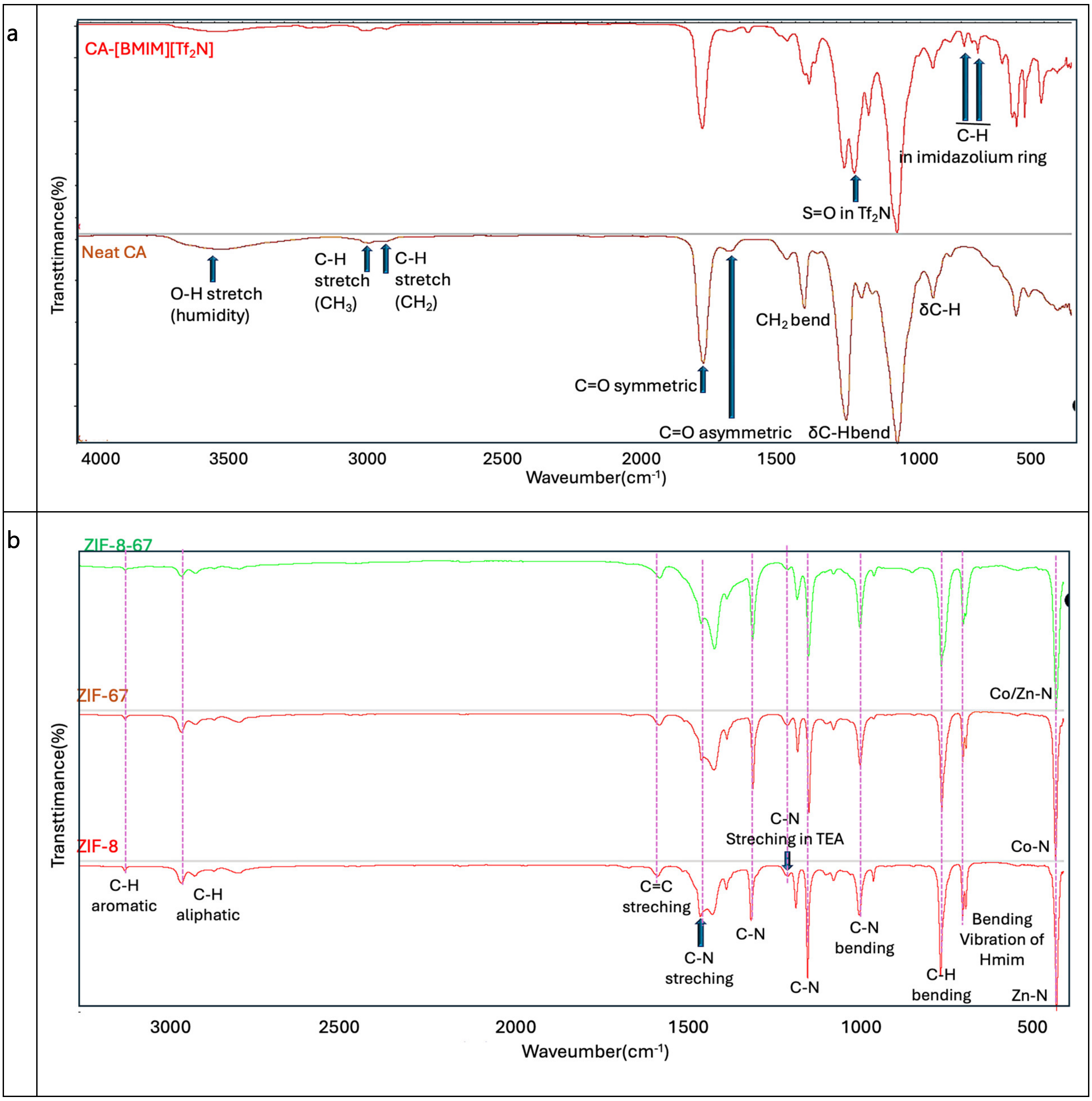

a
CA-[BMIM][Tf2N]
C-H
in imidazolium ring
S=O in Tf2N
Neat CA
O-H stretch
(humidity)
C-H
stretch
(CH3)
C-H
stretch
(CH2)
CH2 bend
δC-H
C=O symmetric
C=O asymmetric
δC-Hbend
Transttimance(%)
4000
3500
3000
2500
2000
1500
1000
500
Waveumber(cm-1)
b
ZIF-8-67
ZIF-67
ZIF-8
Co/Zn-N
C-N
Streching in TEA
Co-N
C-H
aromatic
C-H
aliphatic
C=C
streching
C-N
streching
C-N
C-N
C-N
bending
Bending
Vibration of
Hmim
C-H
bending
Zn-N
Transttimance(%)
3000
2500
2000
1500
1000
500
Waveumber(cm-1)

c

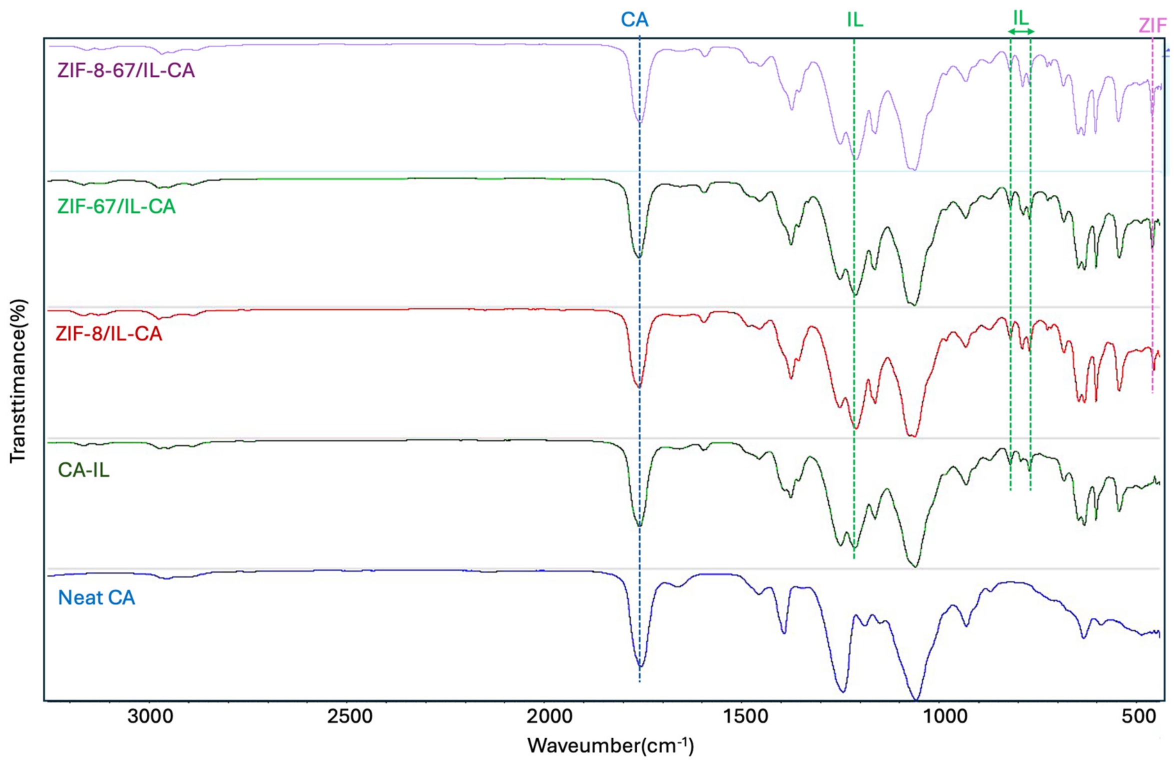

Figure S4. The FTIR spectra of a) neat CA and IL-CA blend-membrane, b) ZIF family, c) ZIF/IL-CA MMMs.

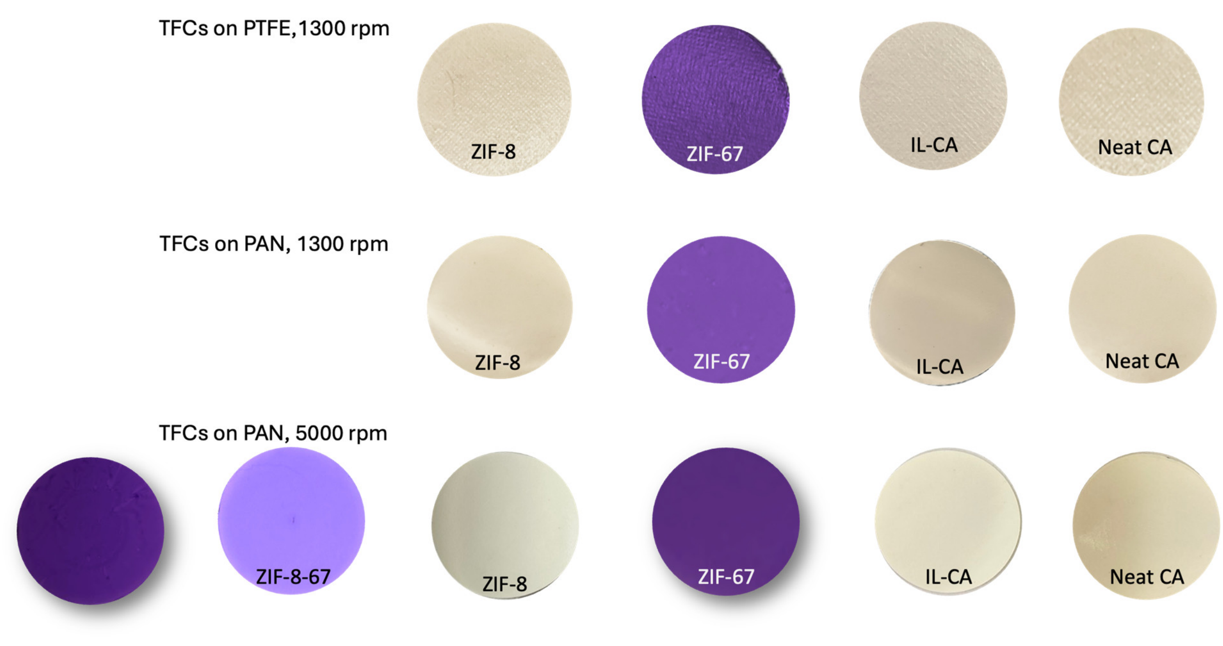

Figure S5. The physical appearance of all fabricated TFCs.

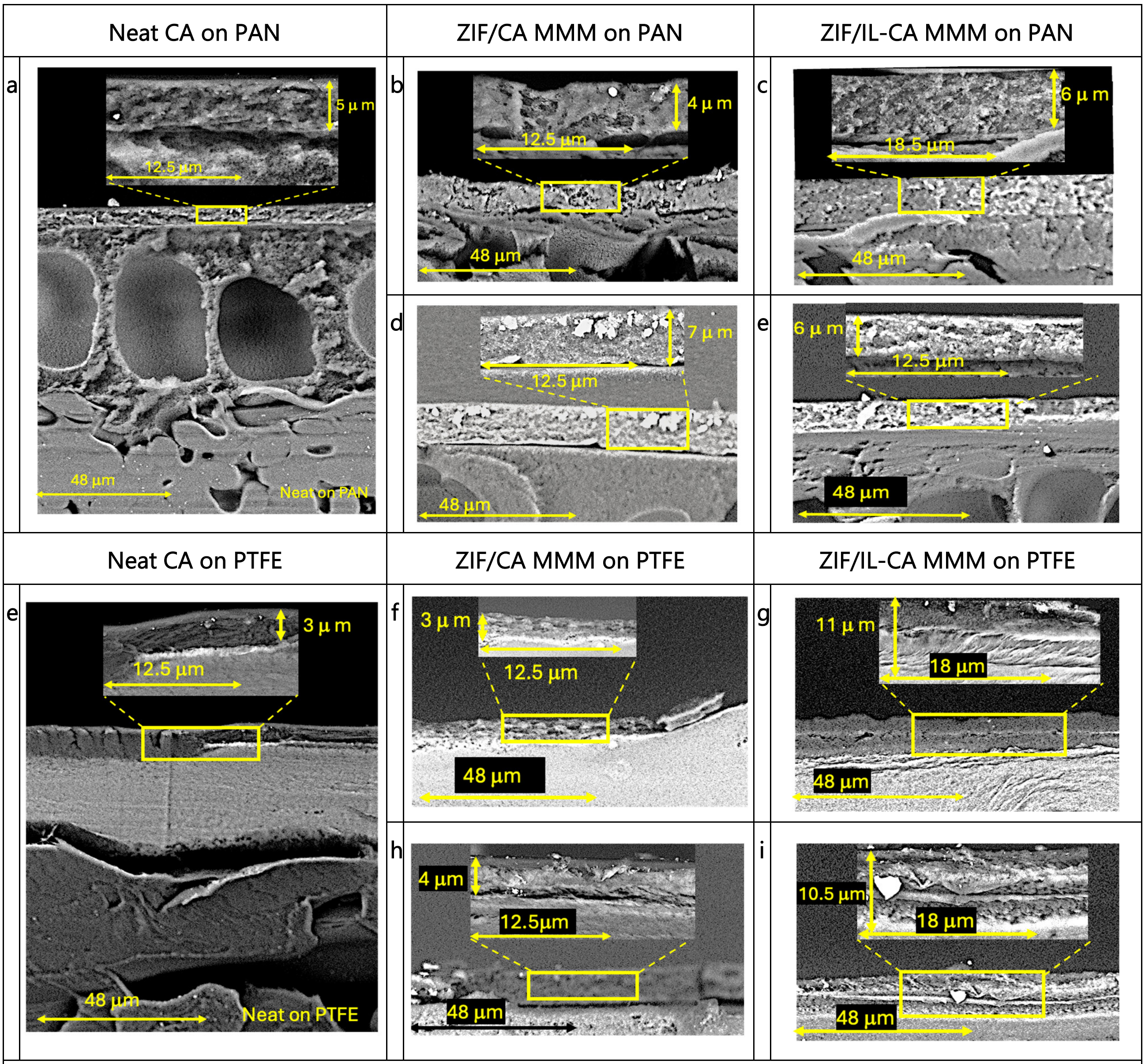

Figure S6. SEM images of MMM TFCs made at 1300 rpm on PAN and PTFE. a,e) neat CA, b,f) ZIF-8/CA, d,h) ZIF-67/CA, c,g) ZIF-8/IL-CA, e,i) ZIF-67/IL-CA at 1300 rpm. "Magnification 1000 x for whole membrane and 2000-3000x for the insert of the thin film when printed on A4 size paper".

SI3. Transport properties

SI3.1. Fixed-volume single-gas Permeation

The time-lag method is based on the solution-diffusion model, and represents the time that a molecule of gas requires to achieve the permeate side, obtainable by recognising the intercept of the linear portion of the pressure-time curve, which is inversely proportional to the effective diffusivity, Eq.SI 1.

$\theta = \frac{L^2}{6D}$	**Eq.SI 1**

The entire permeation curve is expressed by an equation derived from Fick's first and second laws:

$p_t = p_0 + \left(\frac{dp}{dt}\right)_0 \cdot t + \frac{RT}{V_P \cdot V_m} \cdot A \cdot l \cdot p_f \cdot S \times \left(\frac{D \cdot t}{l^2} - \frac{1}{6} - \frac{2}{\pi^2} \sum_{n=1}^{\infty} \frac{(-1)^n}{n^2} \exp\left(-\frac{D \cdot n^2 \cdot \pi^2 \cdot t}{l^2}\right)\right)$	**Eq.SI 2**

Where the derivate p_0 is the starting pressure and $(dp/dt)_0$ *is* the slope of the baseline in the penetration state of the time-lag curve, relating to the eventual presence of micro-defects in the membrane or leaks in the set-up. *R* is the universal gas constant, *T* is the absolute temperature, *A* is the exposed surface area of the membrane, *L* is the thickness, V_p is the permeate volume, V_m is the molar volume of the penetrant gas in standard conditions, p_f is the feed pressure, *S* is the solubility coefficient, and *D* is the diffusion coefficient.

In the stationary state, the exponential tends to zero, and if the baseline slope and the initial pressure are close to zero, Eq.SI 2 can be rearranged as:

$P = \frac{V_P \cdot V_m \cdot l}{RT \cdot A \cdot p_f} \cdot \frac{dp}{dt}$	**Eq.SI 3**

This allows for the direct calculation of the permeability coefficient. Assuming the validity of the solution-diffusion model with pressure and concentration-independent transport parameters, the solubility *S* (m^3_{STP} m^{-3} bar^{-1}) can be determined indirectly from the permeability and the diffusion coefficient:

$S = \frac{P}{D}$	**Eq.SI 4**

The selectivity is calculated as the ratio of the permeability of two different gas species, which is the product of the diffusion selectivity and the solubility selectivity:

$\alpha_P = \frac{P_A}{P_B} = \alpha_D \times \alpha_S = \frac{D_A}{D_B} \times \frac{S_A}{S_B}$	**Eq.SI 5**

SI3.2 Mixed-gas Permeation

The relative sensitivity factor (RS) for each gas is essential for accurate data processing and was determined via a calibration experiment using a mixture of the targeted gas at a known concentration in argon, according to the following equation:

$$RS = \frac{I_i}{I_{Ar}} \cdot \frac{J_{Ar}}{J_i}$$	Eq.SI 6

Where I_i and J_i are the signal intensity and the flow rate for the gas, respectively, while I_{Ar} and J_{Ar} are referred to the signal intensity and the flow rate of Argon. Knowing the sweep flow rate of Argon, one can then determine the unknown flow rate of each permeating gas by:

$$J_i = \frac{p_{P,i}}{p_{P,Ar}} \cdot J_{Ar}$$	Eq.SI 7

Where $p_{P,i}$ and $p_{P,Ar}$ are the partial pressures of the gas of interest and argon, respectively. Consequently, the permeability (P_i) is obtainable as follows:

$$P_i = \frac{J_{p,i}}{(p_{F,i} - p_{P,i})A} . l$$	Eq.SI 8

where A is the membrane area and l is the membrane thickness, while $p_{F,i}$ and $p_{P,i}$ are the partial pressure in feed and permeate sides for each gas.

Employing the well-known time-lag method [55], the diffusion coefficient (D) is determined after integration of the permeate flow rate signal to the total permeate volume as a function of time, Eq. 1. In the present case, with a constant pressure/variable volume, the cumulative volume of permeating gas can be represented as the total permeate volume, $V_{t,STP}$, in time:

$$V_{t,STP} = V_0 + \left(\frac{dV}{dt}\right)_0 \cdot t + \frac{A \cdot p_f \cdot S \cdot D}{l} \cdot \left(t - \frac{l^2}{6D}\right)$$	Eq.SI 9

where the membrane time lag (Θ) is obtained from the intercept between the extrapolated baseline curve (V_0 + $t \cdot (dV/dt)_0$, and of the cumulative permeate volume versus time at steady state. Alternatively, it can also be obtained directly from the appropriate analysis of the sigmoidal permeation curve of the permeate flow rate versus time, without prior integration [7].

a	b

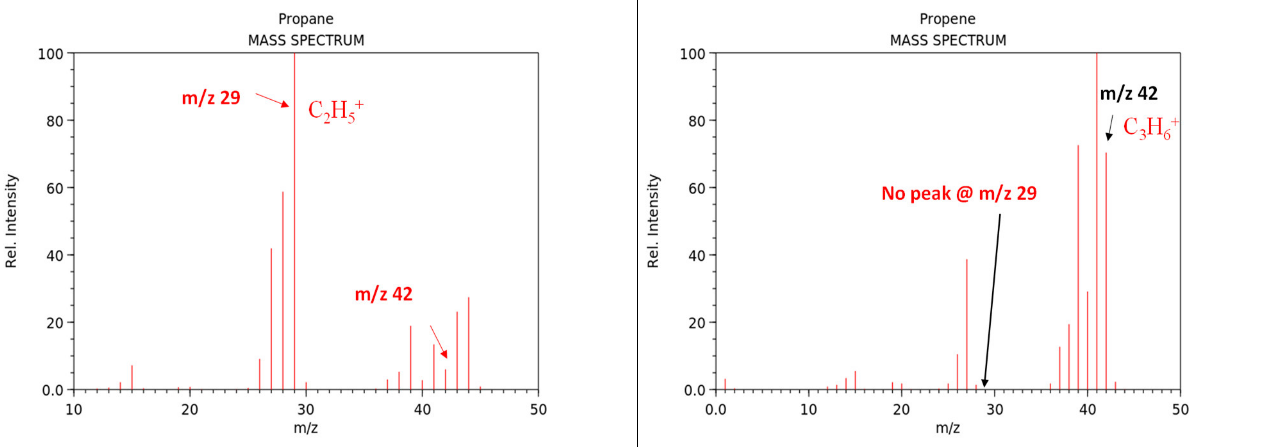

Figure SI 7. Electron ionization (EI) Mass spectrum for (a) propane and (b) propene. *Adapted from NIST Chemistry WebBook Copyright by the U.S. Secretary of Commerce on behalf of the United States of America. (https://webbook.nist.gov/cgi/cbook.cgi?ID=C115071&Units=SI&Mask=200#Mass-Spec, Last accessed on 12/02/2025).*

SI3.3. Single-gas permeation through MMMs on PAN and PTFE

Figure SI 8 shows the overview of the CO_2 permeance and CO_2/CH_4 selectivity of the MMM TFCs, coated on PAN and PTFE at 1300 rpm. The gas permeance of TFCs was normalised for 1µm thickness, and to compare, the CO_2 and CH_4 permeances through the neat free-standing CA membrane [8] were simulated for 1 µm thickness. Moreover, Figure SI 9 and Figure SI 10 illustrate the permeance of CO_2 versus time across different MMM TFCs, providing the opportunity to follow the difference in the permeation behaviours via the time lag method [9].

The results of comparing the neat free-standing membrane with the corresponding TFCs on PAN and PTFE displayed an increase in CO_2 permeance in both the TFCs, accompanied by an improvement in CO_2/CH_4 selectivity of the TFC on PAN but a sharp decrease in that on PTFE (Figure SI 8). This observation, along with a remarkably shorter time lag of the neat TFCs on PAN and PTFE, 15s and ~ 11s, respectively, compared to the free-standing one, 55s, Figure SI 9, suggests an increase in the gas diffusivity as a dominating reason for CO_2 permeance improvement. However, a noticeable decline in CO_2/CH_4 selectivity of the neat TFC on PTFE, compared to that on PAN and that of the free-standing membrane (Figure SI 8), suggests the presence of some defects in this TFC. This hypothesis was confirmed by the presence of a distinguishable baseline slope in the CO_2 permeance curve of the neat CA on PTFE, Figure SI 9, compared to an insignificant slope of that on PAN and a free-standing one.

Table SI 4 reveals the baseline slope calculation and the corresponding %deviation, obtained by applying Eq.SI 2. Compared to the free-standing film, the TFC configuration on PAN generally led to smaller baseline and steady-state slope difference and reduced the related %deviation, as well as keeping this value below 20% for most gases. This indicates an effective isolation of the membrane's selective layer for the gas transport and

minimising the pinhole contributions. Nevertheless, in line with previous observations in Figure SI 8 and Figure SI 9, as well as the SEM images in Figure S6, the baseline slope and %deviation of the membranes on PTFE were relatively higher (%) than those on PAN and CA film. This could be ascribed to the hydrophobic nature of PTFE and its low surface energy [10], incompatible with the hydrophilic CA matrix, resulting in a poor adhesion of neat CA on PTFE, forming unselective interfacial gaps and boosted diffusion of both gases via these gaps, compared to that on PAN and free-standing membranes. Therefore, as Table SI 4 shows, using an appropriate porous support in TFCs plays a crucial role by providing mechanical reinforcement to the thin selective layer, effectively suppressing the propagation of microcracks and facilitating the formation of a defect-free coating. Moreover, TFC fabrication minimises the corresponding time lags, providing a more controlled and homogeneous permeation pathway, attributable to the improved structural integrity of the membranes.

Adding ZIFs, as shown in Figure SI 8, generally increased the CO_2 permeance, accompanied by an improvement in CO_2/CH_4 selectivity of those on PTFE but a decrease in those on PAN. An increase in CO_2 permeance could be explained by the introduction of unselective interfacial gaps between the ZIF NPs and the CA matrix due to their incompatibility, leading to a decline in the ideal selectivity of those on PAN. These unselective areas are observable in the SEM images in Figure S6. The same results were reported in other works [11]. In line with these observations, compared to the neat CA TFC on PAN, the existence of interfacial voids was confirmed by shorter time lags for CO_2 of ZIF/CA,~2s and 3s for ZIF-8/CA and ZIF-67/CA, respectively (Figure SI 10a). Nevertheless, for those ZIF/CA MMMs coated on PTFE, an increase in CO_2 permeance led to an improvement in CO_2/CH_4 selectivity (Figure SI 8), although both the ZIF-8/CA and ZIF-67/CA TFCs presented shorter time lags than the neat TFC (Figure SI 10b). One hypothesis for this could be the role of ZIF NPs in filling the gap between the membrane layer and the PTFE support, as observed in neat CA (Figure SI 8 and Figure SI 9) and in the SEM images (Figure S6. ZIF NPs acted as anchors between the MMMs layer and the PTFE, helping the reduction of unselective voids, which sets a barrier for methane permeance but an increase in that of CO_2 due to the smaller size of the CO_2 molecule and the high affinity of ZIFs to absorb it.

On the other hand, $[BMIM]^+[Tf_2N]^-$ acted as a compatibility enhancer for ZIF/CA MMMs on PAN, confirmed by reduced interfacial gaps and ZIF NPs aggregation, as depicted in SEM images (Figure S6). The resulting ZIF/IL-CA TFCs exhibited lower CO_2 permeance with slightly higher time lags and increased selectivity, supporting the modifying effect of the IL. For ZIF/IL-CA TFCs on PTFE, the plasticiser effect of the IL caused an increase in permeance, confirmed by their shorter CO_2 time lags (Figure SI 10), and a decrease in the ideal selectivity, compared to ZIF/CA MMMs (Figure SI 8). These observations collectively demonstrated the superior performance of the PAN support compared to PTFE, leading the authors to continue the remainder of the study using only PAN-supported TFCs. This choice also aligns with the study’s sustainability goals.

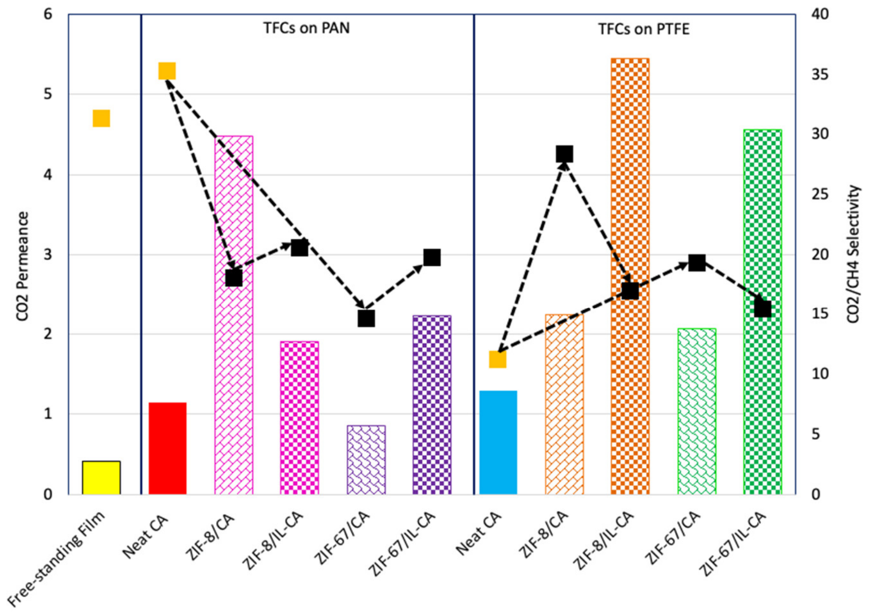

Figure SI 8- The CO_2 permeance and CO_2/CH_4 selectivity through free-standing film and MMM TFCs on PAN and PTFE. The columns indicate the CO_2 permeance. The orange squares show the CO_2/CH_4 selectivity of neat CA membranes and black squares refer to that of MMM TFCs.

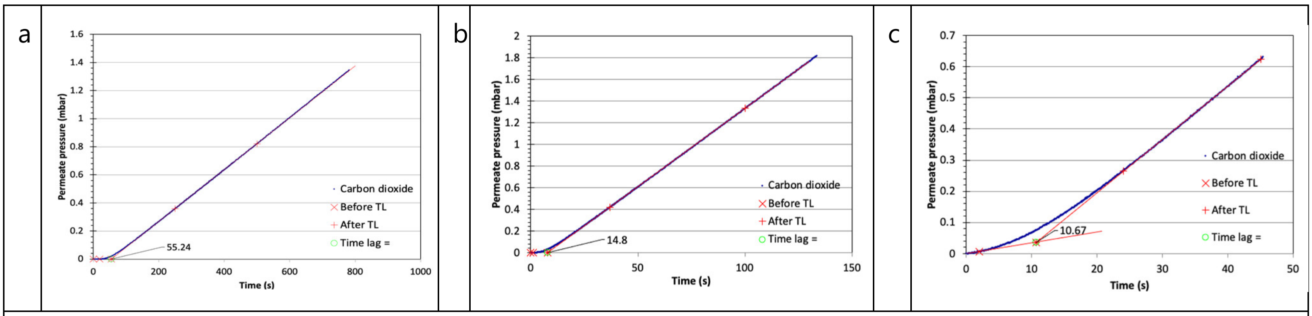

Figure SI 9- CO_2 permeation versus time for neat CA membranes a) free-standing, b) TFC on PAN at 1300 rpm, c) TFC on PTFE at 1300 rpm.

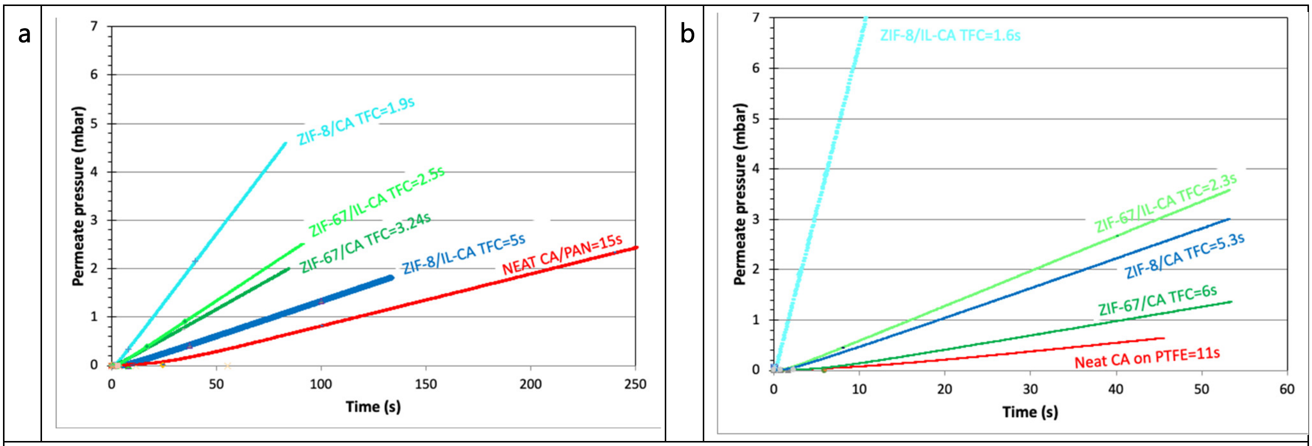

Figure SI 10- CO_2 permeance versus time for TFCs, fabricated at 1300 rpm, on a) PAN and b) PTFE. The values of time lag periods for each TFC are mentioned in seconds. The thickness of TFCs were assumed as 1μm.

Table SI 4. Pinhole effect on neat CA membranes (film and TFC) gas permeability measurement.

Neat CA membrane	Gas	Steady-state slope (mbar/s)	Fitted baseline Slope (mbar/s)	Deviation (%)	Intercept (mbar)	Baseline Flow Rate (cm^3/min)	Corrected Flow Rate (cm^3/min)	time lag (s)
Film		1.74×10^{-4}	2.0×10^{-5}	11.5	1.4×10^{-3}	2.75×10^{-2}	0.24	34.6
TFC on PAN	N_2	4.04×10^{-4}	3.09×10^{-4}	76.5	1.0×10^{-3}	4.26×10^{-1}	0.56	5.69
TFC on PTFE		1.39E-03	3.10×10^{-4}	22.2	9.53×10^{-4}	4.27×10^{-1}	1.50	8.12
Film		1.71×10^{-4}	2.4×10^{-5}	14.2	9.0×10^{-4}	3.33×10^{-2}	0.20	163
TFC on PAN	CH_4	4.21×10^{-4}	6.55×10^{-5}	15.5	6.89×10^{-4}	9.04×10^{-4}	0.49	27.5
TFC on PTFE		3.23×10^{-3}	1.83×10^{-3}	56.6	1.6×10^{-4}	2.52	1.94	10.31
Film		1.85×10^{-3}	6.4×10^{-5}	3.5	2.0×10^{-4}	2.51×10^{-1}	7.22	55.2
TFC on PAN	CO_2	1.45×10^{-2}	4.97×10^{-4}	3.4	2.56×10^{-4}	6.86×10^{-1}	19.96	14.8
TFC on PTFE		1.71×10^{-2}	3.45×10^{-3}	20.2	2.16×10^{-4}	4.76	18.84	10.67
Film		3.67×10^{-5}	1.1×10^{-5}	30.7	2.8×10^{-4}	4.40×10^{-2}	0.1	7508
TFC on PAN	C_3H_6	8.01×10^{-5}	7.0×10^{-6}	9.4	6.32×10^{-4}	2.93×10^{-2}	0.28	1600
TFC on PTFE		3.54×10^{-3}	1.82×10^{-3}	51.5	1.39×10^{-3}	7.13	6.71	15.1
Film		0.6×10^{-5}	1.31×10^{-5}	45.3	1.5×10^{-3}	2.33×10^{-2}	3×10^{-2}	15649
TFC on PAN	C_3H_8	7.36×10^{-6}	2.62×10^{-5}	28.1	5.30×10^{-4}	2.88×10^{-2}	7×10^{-2}	5856
TFC on PTFE		3.49×10^{-3}	1.07×10^{-3}	30.6	5.23×10^{-4}	4.17	9.47	17.41

Table SI 5. Gas transport properties of ZIF/IL-CA TFC MMMs on PAN at 5000 rpm.

Membrane	Gas					(Sorption/Diffusion) selectivity		
	C_3H_6	C_3H_8	N_2	CO_2	CH_4			
Neat CA TFC	D_A [10^{-12} m^2s^{-1}]					D_{C3H6}/D_{C3H8}	D_{CO2}/D_{CH4}	D_{CO2}/D_{N2}
	$3.95E^{-04}$	$1.17E^{-04}$	$2.93E^{-02}$	$2.09E^{-02}$	$8.07E^{-03}$	3.36	2.59	0.71
	S_A [$cm^3_{(STP)}cm^{-3}$ bar^{-1}]					S_{C3H6}/S_{C3H8}	S_{CO2}/S_{CH4}	S_{CO2}/S_{N2}
	30.2	26.3	0.8	41.0	3.0	1.15	13.64	50.49
IL-CA TFC	D_A [10^{-12} m^2s^{-1}]					D_{C3H6}/D_{C3H8}	D_{CO2}/D_{CH4}	D_{CO2}/D_{N2}
	$3.53E^{-04}$	$1.52E^{-04}$	$1.99E^{-02}$	$1.26E^{-02}$	$9.67E^{-03}$	2.32	1.30	0.63
	S_A [$cm^3_{(STP)}cm^{-3}$ bar^{-1}]					S_{C3H6}/S_{C3H8}	S_{CO2}/S_{CH4}	S_{CO2}/S_{N2}
	$7.20E^{+01}$	$2.58E^{+01}$	$8.62E^{-01}$	$4.41E^{+01}$	1.98	2.79	22.25	51.16
ZIF-8/IL-CA TFC	D_A [10^{-12} m^2s^{-1}]					D_{C3H6}/D_{C3H8}	D_{CO2}/D_{CH4}	D_{CO2}/D_{N2}
	$1.72E^{-03}$	$6.86E^{-04}$	$4.77E^{-02}$	$4.91E^{-02}$	$1.70E^{-02}$	2.51	2.89	1.03
	S_A [$cm^3_{(STP)}cm^{-3}$ bar^{-1}]					S_{C3H6}/S_{C3H8}	S_{CO2}/S_{CH4}	S_{CO2}/S_{N2}
	72.6	26.3	1.0	29.1	2.5	2.76	6.98	28.61
ZIF-67/IL-CA TFC	D_x [10^{-12} m^2s^{-1}]					D_{C3H6}/D_{C3H8}	D_{CO2}/D_{CH4}	D_{CO2}/D_{N2}
	$1.09E^{-03}$	$1.87E^{-04}$	$7.34E^{-02}$	$1.10E^{-01}$	$3.54E^{-02}$	5.81	3.11	1.50
	S_A [$cm^3_{(STP)}cm^{-3}$ bar^{-1}]					S_{C3H6}/S_{C3H8}	S_{CO2}/S_{CH4}	S_{CO2}/S_{N2}
	37.0	23.4	0.7	15.7	1.8	1.58	8.94	21.78
ZIF-8-67/IL-CA	D_x [10^{-12} m^2s^{-1}]					D_{C3H6}/D_{C3H8}	D_{CO2}/D_{CH4}	D_{CO2}/D_{N2}
	$1.22E^{-03}$	$2.19E^{-04}$	$9.00E^{-02}$	$1.00E^{-01}$	$3.66E^{-02}$	5.55	2.73	1.11
	S_A [$cm^3_{(STP)}cm^{-3}$ bar^{-1}]					S_{C3H6}/S_{C3H8}	S_{CO2}/S_{CH4}	S_{CO2}/S_{N2}
	29.4	15.2	0.5	16.1	1.6	1.94	10.41	29.53

Table SI 6. transport parameters of TFC membrane ZIF-8-67/IL-CA						
	Permeance (GPU)				Time lag (s)	
	Pure gas	Mixed gases			Pure gas	Mixed gases
C_3H_6	0.0512	0.047 P↘		C_3H_6	80.3	38.8 D↗
C_3H_8	0.0069	0.0072 P↗		C_3H_8	450	334 D↗
α_P *)	7.4	6.5 α↘		α_D **)	5.6	8.6 α↗

*) $\alpha_P = \frac{P_{C3H6}}{P_{C3H8}}$ **) $\alpha_D = \frac{D_{C3H6}}{D_{C3H8}} = \frac{\Theta_{C3H8}}{\Theta_{C3H6}}$

SI Bibliography

1. Gross, A.F.; Sherman, E.; Vajo, J.J. Aqueous Room Temperature Synthesis of Cobalt and Zinc Sodalite Zeolitic Imidizolate Frameworks. *Dalton transactions* 2012, *41*, 5458–5460.
2. Pan, Y.; Liu, Y.; Zeng, G.; Zhao, L.; Lai, Z. Rapid Synthesis of Zeolitic Imidazolate Framework-8 (ZIF-8) Nanocrystals in an Aqueous System. *Chemical Communications* 2011, *47*, 2071–2073.
3. Nordin, N.; Ismail, A.F.; Mustafa, A.; Goh, P.S.; Rana, D.; Matsuura, T. Aqueous Room Temperature Synthesis of Zeolitic Imidazole Framework 8 (ZIF-8) with Various Concentrations of Triethylamine. *RSC Adv* 2014, *4*, 33292–33300.
4. Chang, P.-H.; Lee, Y.-T.; Peng, C.-H. Synthesis and Characterization of Hybrid Metal Zeolitic Imidazolate Framework Membrane for Efficient H2/CO2 Gas Separation. *Materials* 2020, *13*, 5009.
5. Li, J.; Chang, H.; Li, Y.; Li, Q.; Shen, K.; Yi, H.; Zhang, J. Synthesis and Adsorption Performance of La@ ZIF-8 Composite Metal–Organic Frameworks. *RSC Adv* 2020, *10*, 3380–3390.
6. Guo, X.; Xing, T.; Lou, Y.; Chen, J. Controlling ZIF-67 Crystals Formation through Various Cobalt Sources in Aqueous Solution. *J Solid State Chem* 2016, *235*, 107–112.
7. Monteleone, M.; Fuoco, A.; Esposito, E.; Rose, I.; Chen, J.; Comesaña-Gándara, B.; Bezzu, C.G.; Carta, M.; McKeown, N.B.; Shalygin, M.G. Advanced Methods for Analysis of Mixed Gas Diffusion in Polymeric Membranes. *J Memb Sci* 2022, *648*, 120356.
8. Hajivand, P.; Longo, M.; Mastropietro, T.F.; Godbert, N.; Monteleone, M.; Armentano, D.; Jansen, J.C. Tailoring the Thermal, Mechanical, and Gas Transport Properties of Cellulose Acetate Membranes with Ionic Liquids for Efficient Propene/Propane Separation. *Carbohydr Polym submitted*.
9. Fraga, S.C.; Monteleone, M.; Lanč, M.; Esposito, E.; Fuoco, A.; Giorno, L.; Pilnáček, K.; Friess, K.; Carta, M.; McKeown, N.B. A Novel Time Lag Method for the Analysis of Mixed Gas Diffusion in Polymeric Membranes by On-Line Mass Spectrometry: Method Development and Validation. *J Memb Sci* 2018, *561*, 39–58.
10. Xiong, S.; Jia, X.; Mi, K.; Wang, Y. Upgrading Polytetrafluoroethylene Hollow-Fiber Membranes by CFD-Optimized Atomic Layer Deposition. *J Memb Sci* 2021, *617*, 118610.
11. Vu, M.-T.; Lin, R.; Diao, H.; Zhu, Z.; Bhatia, S.K.; Smart, S. Effect of Ionic Liquids (ILs) on MOFs/Polymer Interfacial Enhancement in Mixed Matrix Membranes. *J Memb Sci* 2019, *587*, 117157.
